# Phototoxicity of Tridentate Ru(II) Polypyridyl Complex with Expanded Bite Angles toward Mammalian Cells and Multicellular Tumor Spheroids

**DOI:** 10.1021/acs.inorgchem.3c01982

**Published:** 2023-08-03

**Authors:** Rhianne C. Curley, Christopher S. Burke, Karmel S. Gkika, Sara Noorani, Naomi Walsh, Tia E. Keyes

**Affiliations:** † School of Chemical Sciences and National Centre for Sensor Research, Dublin City University, Dublin 9 D09 NA55, Ireland; ‡ National Institute for Cellular Biotechnology, School of Biotechnology, Dublin City University, Dublin 9 D09 NA55, Ireland

## Abstract

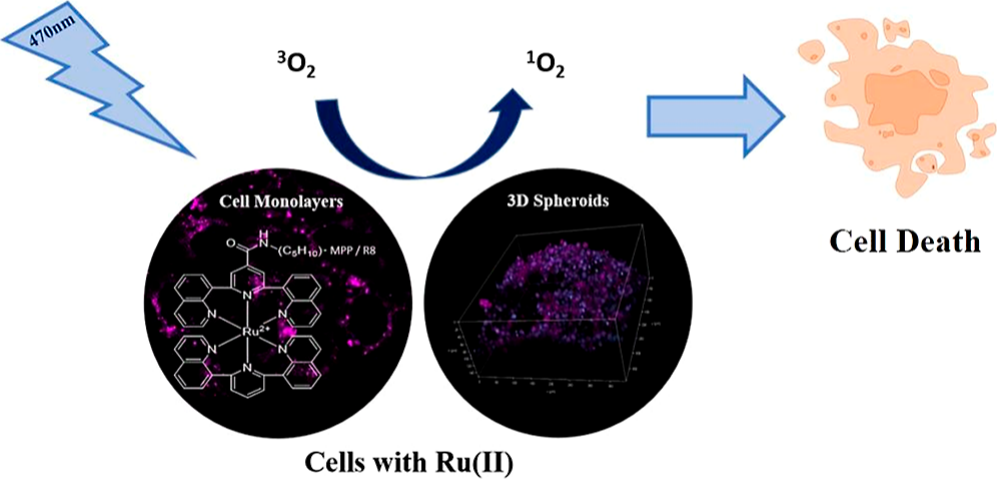

Tridentate ligand-coordinated ruthenium (II) polypyridyl complexes with large N–Ru–N bite angles have been shown to promote ligand field splitting and reduce singlet–triplet state mixing leading to dramatically extended emission quantum yields and lifetimes under ambient conditions. These effects are anticipated to enhance their photoinduced singlet oxygen production, promoting prospects for such complexes as type II phototherapeutics. In this contribution, we examined this putative effect for [Ru(bqp)(bqpCOOEt)]^2+^, Ru-bqp-ester, a heteroleptic complex containing bqp = [2,6-bi(quinolin-8-yl)pyridine], a well-established large bite angle tridentate ligand, as well as its peptide conjugates [Ru(bqp)(bqpCONH-ahx-FrFKFrFK(Ac)-CONH_2_)]^5+^ (Ru-bqp-MPP) and [Ru(bqp) (bqp)(CONH-ahx-RRRRRRRR-CONH_2_)]^10+^ (Ru-bqp-R8) that were prepared in an effort to promote live cell/tissue permeability and targeting of the parent. Membrane permeability of both parent and peptide conjugates were compared across 2D cell monolayers; A549, Chinese hamster ovary, human pancreatic cancer (HPAC), and 3D HPAC multicellular tumor spheroids (MCTS) using confocal microscopy. Both the parent complex and peptide conjugates showed exceptional permeability with rapid uptake in both 2D and 3D cell models but with little distinction in permeability or distribution in cells between the parent or peptide conjugates. Unexpectedly, the uptake was temperature independent and so attributed to passive permeation. Both dark and photo-toxicity of the Ru(II) complexes were assessed across cell types, and the parent showed notably low dark toxicity. In contrast, the parent and conjugates were found to be highly phototoxic, with impressive phototoxic indices (PIs) toward HPAC cell monolayers in particular, with PI values ranging from ∼580 to 760. Overall, our data indicate that the Ru(II) parent complex and its peptide conjugates show promise at both cell monolayers and 3D MCTS as photosensitizers for photodynamic therapy.

## Abstract 2

Parent and peptide conjugates of bistridentate Ru(II) complexes with expanded bite angles show high phototoxicity toward cancerous and non-cancerous cell monolayers, and pancreatic multicellular tumor spheroids upon irradiation at 470 nm.

## Introduction

Photodynamic therapy (PDT) or photosensitized light therapy is a long standing medical treatment that has been applied to the treatment of cancers as well as infection and skin conditions.^1,2^ The most common mode of application is that the photosensitizer (PS), under illumination by light of non-tissue destructive wavelengths, in the visible or near-infrared range (typically 630–800 nm), initiates the formation of singlet oxygen from ambient oxygen at the site of treatment to destroy the targeted cells.^3,4^ In biological environments, singlet oxygen species typically have limited lifetimes (<3 μs), with an estimated half-life of approximately 40 ns and a diffusive distance of <268 nm.^5−7^ Meaning the sensitizer must permeate the site of therapy. This format of PDT is known as type II PDT and constitutes the mechanism behind the majority of clinically approved PS for PDT, which have been based on tetrapyrrolic structures, for example, porphyrin, chlorin, and phthalocyanine.^8−11^ Photofrin, for example, is a clinically approved PS hematoporphyrin derivative used for PDT treatment of, but not limited to, lung and esophageal cancer.^12^ Many organic PS species traditionally applied to PDT have a number of drawbacks, including poor aqueous solubility, propensity for aggregation, skin photosensitivity, and dark toxicity.^13,14^ There is, therefore, demand for the development of new classes of PS that address these issues. An ideal PS should be amphiphilic, display good solubility in biological media or water and exhibit minimum toxicity in the dark, but high toxicity when activated by a light source. Additionally, PS should be photostable given the short diffusive distances of singlet O_2_, they should be cell and tumor permeable.^15^ Luminescent co-ordination compounds, such as those of ruthenium, are potentially very attractive agents for PDT as they are constitutionally triplet state emitters with high extinction coefficients (minimizing the PS dosage required) and have versatile chemistry that can be used to tune the properties of the compound to meet the demanding criteria of a PS. As they are usually charged, they tend not to self-aggregate into structures that diminish their photophysical properties or solubility and they have favorable photophysical properties, including good photostability.^16^

Metal polypyridyl luminophores are consequently under intensive investigation across a range of biological applications, including as luminescent probes for intracellular imaging, sensing,^17,18^ as well as for their phototherapeutic potential in PDT.^19^ Ru(II) complexes in particular are under study as sensitizers for routine type-II PDT applications, and type-II Ru(II) sensitizers have been rationally designed for 2-photon PDT.^20−23^ TLD1433, a polypyridyl Ru(II) complex designed for the treatment of non-muscle-invasive bladder cancer (NMIBC), was the first Ru(II)-based PDT agent to enter a human clinical trial.^24^ TLD1433 is administered through bladder instillation 1 h prior to light treatment and demonstrates high retention in bladder cancer cells.^25^ Furthermore, Chamberlain et al. reported the successful destruction of A549 lung cancer cells in vitro upon irradiation of TLD1433 at 532 and 630 nm.^26^

As their excited state is formally a triplet, Ru(II) polypyridyl complexes, in oxygenated environments can undergo Dexter, triplet–triplet energy transfer with molecular oxygen to form tissue-destructive singlet oxygen. However, the metal-to-ligand charge-transfer (^3^MLCT) state is short-lived relative to organic phosphors because the heavy ruthenium atom tends to mix singlet and triplet character in the MLCT state. Consequently, they tend to be less efficient singlet oxygen sensitizers than metal porphyrins, for example, whose PS efficiency is well established.^27^ But, Ru(II) complexes owing to their synthetic versatility, can be tailored to offer the aforementioned advantages of lower dark cytotoxicity and cell targeting.^28,29^ Means to promote singlet oxygen sensitization in such complexes include increasing the triplet character of the excited state, e.g., through mixing ^3^LC states with the ^3^MLCT state, by reducing singlet–triplet mixing of the ^3^MLCT state or by enhancing the ^3^MLCT lifetime. The latter is accomplished by manipulation of the relative energy of the triplet metal-centered state whose thermal population, likely to be promoted in the warm cellular environment, increases non-radiative decay. This has been achieved in practice, by increasing the energy gap between the ^3^MLCT and ^3^MC through tuning the σ donor and π accepting abilities of the ligands.^30^ Moreover, in the particular case of tridentate ligand coordinated ruthenium (II) polypyridyl complexes, it has been very effectively accomplished by expanding the N–Ru–N bite angles of the coordination cage.^31,32^ The resulting release of steric strain facilitates a less distorted octahedral geometry and has been shown to dramatically extend emission quantum yield and lifetime under ambient conditions.^33−35^ In an example by Schubert et al., it was demonstrated that both approaches can be combined to profoundly lengthen the ^3^MLCT excited state.^36^

A key challenge in the biological application of luminescent coordination compounds, such as ruthenium, osmium, and platinum, is membrane permeability and subcellular localization. Non-specific localization can result in the activation of the phototherapeutic effects off-target, and thus, in PDT applications, damage to the surrounding cells.^37,38^ The mechanism of cellular uptake of ruthenium complexes can vary depending on complex properties, such as its size, charge, and lipophilicity.^39^ Increasing the lipophilicity of a ruthenium complex should increase the uptake via passive diffusion as was demonstrated by Park and co-workers.^40^ However, the mechanism of uptake can also depend on the concentration of the complex administered and the gene expression of the cell line, which can upregulate proteins, such as efflux pumps and proteins for active transport.^41,42^ Cell penetrating peptides (CPPs), such as octa-arginine sequences (RRRRRRRR), and signal peptides, such as mitochondrial penetrating peptides (MPP), have been proven effective in promoting permeation and targeting of ruthenium polypyridyl complexes in cells but less is known about their effectiveness in promoting uptake and permeation through tissues.^39^ Peptide vectorization has been successfully exploited by our group and others to target metal complex luminophores to specific organelles for imaging and sensing.^43,44^ For example, we previously reported a peptide-bridged dinuclear Ru(II) conjugate [(Ru-(bpy)_2_phen-Ar)_2_-MPP],^7+^ which allowed for precise targeting to the mitochondria. More recently, we demonstrated an octa-arginine-conjugated osmium(II) complex [Os-(R4)_2_]^10+^ that successfully penetrated 3D pancreatic cancer tumor spheroids.^45,46^

Although 2D cell monolayers offer a convenient and well-accepted approach for evaluating permeation in drug development, 3D cellular models, such as spheroids and cellular aggregates, provide a more physiologically relevant cellular environment.^47^ The layered structure of spheroids mimics the tumor microenvironment more closely than 2D cell monolayers. Nutrient and oxygen gradients in spheroids mimic that of tumors in vivo with the vast majority of spheroids comprising three zones, the outer proliferative zone, the senescent zone, and the necrotic (hypoxic) core.^48^ Increased cell-to-cell and cell-to-extracellular matrix interactions lead to enhanced physical barriers in spheroids that make drug permeation more difficult, which better reflects drug uptake in vivo.^49^ In the last decade, 3D multicellular spheroids and cell aggregates have become increasingly popular models for drug discovery and function as an excellent intermediate model between in vitro cell monolayers and in vivo tumors. Imaging, sensing, and therapeutic evaluation in 3D cell models requires luminescent probes that can deeply penetrate a spheroid or cellular aggregate. Significant differences between the permeation and destination of luminescent probes between 2D and 3D cellular models have been reported.^50^ Therefore, in assessing potential PS sensitizers, permeation through 3D models should ideally also be considered but rarely have been to date in metal complex species, with only a small number of studies reported to date assessing metal complex PSs in spheroids.^51,52^ A recent work includes lysosomal localizing Ru (II) complexes, studied by the Chao group in HeLa spheroids for two photon PDT, irradiating the spheroids at 10 J/cm^2^ at 800 nm, and also the study of photoactive iridium (III) complexes in A549 spheroids by the Sadler group.^53,54^

**Figure 1:**
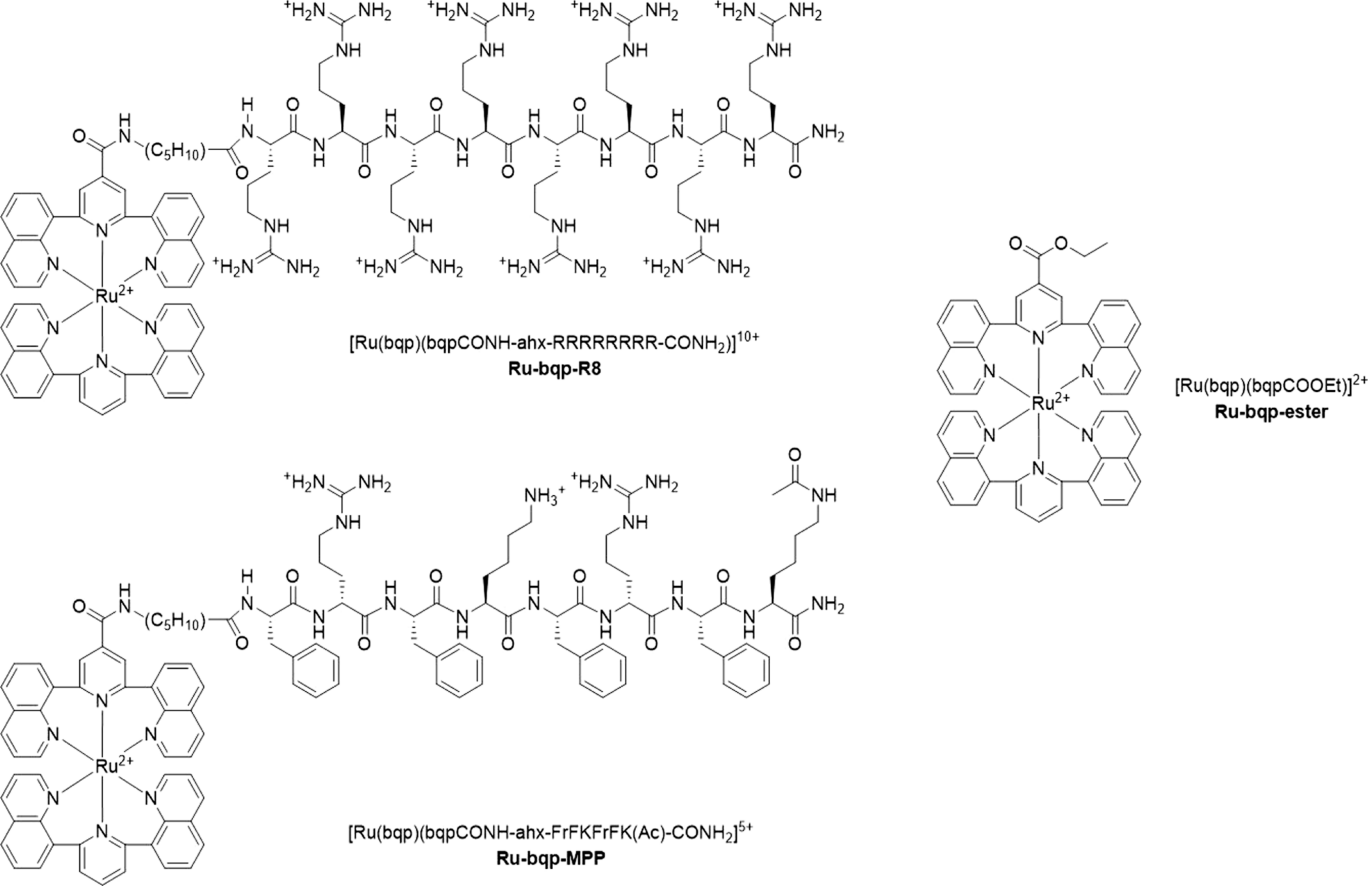
Chemical structures of the complexes and conjugates reported in this work; Ru-bqp-ester, Ru-bqp-R8, and Ru-bqp-MPP.

Herein, we report on the application of tridentate coordinated [Ru(bqp)(bqpCOOEt)]^2+^ (Ru-bqp-ester), where bqp = 2,6-bi(quinolin-8-yl)pyridine, and its peptide conjugates [Ru(bqp)(bqpCONH-ahx-FrFKFrFK(Ac)-CONH_2_)]^5+^ (Ru-bqp-MPP) and [(Ru(bqp)(bqp)CONH-ahx-RRRRRRRR-CONH_2_)]^10+^ (Ru-bqp-R8) as potential phototoxic agents. The parent complex (Figure 1), was selected as, when first reported by Hammarström et al., it showed an exceptionally long lived ^3^MLCT excited state facilitated by the expanded coordination bite angle of the 2,6-bi(quinolin-8-yl)pyridine ligand so that the coordination geometry around the metal center is approximately octahedra.^33,55^ This has the effect of increasing the ligand field of the complex limiting thermal access to the ^3^MC state but studies have also shown there is a dual effect where the ligand also reduces singlet mixing of the triplet excited state, which also contributes to the long lifetime.^56^ We rationalized that both effects should yield a complex that is a good singlet oxygen sensitizer, which is why we selected it for study here. To promote cell membrane permeation, we conjugated the parent complex to peptide and the parent and peptide conjugates were investigated here in live cell monolayers and 3D cancer models. We examined the impact of the peptide conjugation on the photophysical behavior, cell uptake, and dark and phototoxicity of the complex in live, A549 lung cancer, human pancreatic adenocarcinoma (HPAC), Chinese hamster ovary (CHO) non-cancer cell monolayers, and also in 3D HPAC tumor spheroids, where we observe, consistent with the long-lived excited triplet state of the complex, high phototoxicity.

## Experimental Section

### Materials

All chemicals and reagents, DMEM/F-12 Ham, RPMI, and DMEM cell culture media, and corresponding components were purchased from Sigma-Aldrich and were used as received. Phenol red free media (Gibco), fetal bovine serum (FBS, heat-inactivated, Gibco), alamarBLUE reagent (Invitrogen), and co-localizing dyes were purchased from Thermo Fisher Scientific. For synthesis, anion metathesis using tetrabutylammonium chloride in acetone provided the chloride salts from their hexafluorophosphate salts. Peptides were purchased from Celtek Peptides (USA) at >95% purity. All other materials were obtained from Sigma-Aldrich (Merck) or Fluorochem (UK) and were used without further purification.

### Synthesis

The syntheses of bqpCOOEt, mer-[Ru(bqp)(CH_3_CN)_3_](PF_6_)_2_, mer-[Ru(bqp)(bqpCOOH)](PF_6_)_2_, and mer-[Ru(bqp)(bqpCOOEt)](PF_6_)_2_ have been described previously.^57−59^ In this work, an alternate route to mer-[Ru(bqp)(bqpCOOH)](PF_6_)_2_ and mer-[Ru(bqp)(bqpCOOEt)](PF_6_)_2_ was developed (see the Supporting Information). Structure and purity were confirmed by NMR and mass spectra data. ^1^H NMR spectra were recorded at 400 or 600 MHz as indicated using Bruker spectrometers and deuterated solvents for a homo-nuclear lock. The spectra were processed using Bruker Topspin software and were calibrated against solvent peaks according to the published values. High-resolution mass spectrometry (HR-MS) was performed at the HR-MS facility, Trinity College Dublin (parent complexes) or the Mass Spectrometry Facility, NUI Maynooth (conjugates).

### Photophysical Measurements

Absorbance measurements of the Ru(II) complexes at 20 μM were performed using a Jasco V670 spectrophotometer. Emission spectra were collected using a Varian Cary Eclipse fluorescence spectrophotometer and luminescent lifetime measurements were performed on a PicoQuant FluoTime 100 FLS TCSPC system using a 450 nm pulsed laser and an external pulse generator. Lifetime decay curves were analyzed using the PicoQuant Fluofit software with fitting criteria; 0.9 < *X*^2^ < 1.1. For O_2_ sensitivity studies, the emission spectrum and luminescent lifetime of each complex (10 μM) were recorded under O_2_ saturation, and after N_2_ purge for 20 min. All measurements were performed in triplicate and reported as the mean ± SD.

### Cell Monolayer and 3D Spheroid Cell Culture

Three cell lines were selected for cell monolayer studies: a lung carcinoma cancer cell line (A549), a HPAC cell line, and a non-cancer cell line derived from CHO-K1. A 50% Dulbecco’s modified Eagle’s medium (DMEM) and F-12 Nutrient Mixture Ham (F-12 Ham) media was used to culture CHO cells, DMEM for A549 cells, and Rosewell Park Memorial Institute (RPMI-1640) medium for HPAC. Both CHO and A549 media were supplemented with 10% FBS and 1% penicillin–streptomycin, HPAC media were supplemented with 5% FBS. All cells were grown at 37 °C with 5% CO_2_ and sub-cultured at 80–90% confluency. To prepare 3D multicellular tumor spheroids (MCTSs), HPAC cells were seeded at 2 × 10^4^ cells/well in clear U-bottom 96 well plates coated with polyHEMA (poly2-hydroxyethyl methacrylate, Sigma), centrifuged at 700 rpm for 10 min at 37 °C, and allowed to grow and compact for 96 h prior to treatment. A partial media change was performed after 48 h.

### Dark Toxicity Studies

The alamar blue assay (Invitrogen) was used to assess the viability of A549, CHO, and HPAC cell monolayers treated with the Ru(II) probes, Ru-bqp-ester, Ru-bqp-MPP, Ru-bqp-R8. CHO, A549, and 2D HPAC cells were seeded in a 96-well plate (Nunc, flat bottom cell culture treated) at 1 × 10^4^ cells per well, i.e., in 100 μL media for 24 h at 37 °C with 5% CO_2_. Ru-bqp-ester, Ru-bqp-R8, and Ru-bqp-MPP were added for 24 h at 37 °C and 5% CO_2_ in the absence of light. The alamar blue (Resazurin) assay was performed to measure cell viability by replacing the test solution with 100 μL of a 10% v/v media/resazurin solution and incubating for 5 h at 37 °C in the absence of light. Absorbance was measured using a BMG Labtech ClarioStar (plus) plate reader at 570 and 600 nm (corrected for background subtraction). Cell viability is presented as a percentage (%) compared to untreated control wells.

### Phototoxicity Studies

All cell monolayers were prepared as described above for the phototoxicity assay. A549 and CHO cells were treated at varying concentrations of the complexes and incubated for 30 min in the absence of light, HPAC were incubated for 2 h to allow uptake. A 470 nm blue light LED array was used to irradiate the cells in the wells at a total dose of 17 ± 1.64 J/cm^2^ (2 h at 2.37 ± 0.23 mW/cm^2^). A dark control plate was performed alongside all phototoxicity plates. Irradiation conditions were tested on untreated cells to confirm cells remained viable at this dose before any experiments were performed and each phototoxicity plate included untreated test wells for cell viability comparison. All viability assays were performed in triplicate.

### In Vitro Singlet Oxygen Detection

To assess singlet oxygen (^1^O_2_) production and confirm the phototoxicity of our Ru(II) complexes was caused by the generation of reactive oxygen species (ROS), a ^1^O_2_ scavenger study was completed using 1,3-diphenylisobenzofuran (DPBF). As DPBF has poor solubility in aqueous solutions, a stock solution of 10 mM DPBF and 20 μM of the parent complex Ru-bqp-ester was prepared in ethanol with 1% DMSO. Aliquots of 200 μL of this solution were added to a 96 well-plate and irradiated with a 470 nm LED at 2.37 ± 0.23 mW/cm^2^ in 1 min intervals for a total of 6 min and a plate reader was used to measure the absorbance of the solution following each irradiation step. The % change in absorbance was calculated using the formula 100 – [(*X*/*A*) × 100] where *A* = the absorbance of Ru-DPBF at 410 nm where *t* = 0 and *X* = the absorbance at 410 nm at *t* = 1, 2, 3, 4, 5, or 6 min.

### Confocal Laser Scanning Microscopy

Uptake studies were carried out for A549, HPAC, and CHO cell lines. Cells were seeded in 35 mm glass bottom dishes or 4-chamber slides (Ibidi, Germany) at 1 × 10^5^ cells per mL for 24 h at 37 °C at 5% CO_2_. A final concentrations of 30 μM Ru-bqp-ester, Ru-bqp-R8, and Ru-bqp-MPP in growth media were added to the cells and incubated for 1 h in A549 and CHO cells and 4 h in HPAC cells. The dye/media solution was removed and the cells were washed twice with 1× PBS. Cells were imaged in supplemented PBS (MgCl_2_ and CaCl_2_) or phenol red free media. Cells were imaged using a Leica TCS DMi8 inverted confocal microscope with a 63× oil immersion lens and heated stage at 37 °C. The Ru(II) complexes were excited using a 490 nm white light laser and the emission collected at 580–730 nm unless stated otherwise. DRAQ7 (3 μΜ) was added to the cells during initial uptake studies to detect cell death or damage. DRAQ7 is a nuclear dye that is taken up by damaged or dead cells and was excited at 633 nm with emission collected at 680–750 nm.^60^ Temperature-dependent uptake studies were performed using the same protocol described for routine uptake studies at 37 °C, however, the 1 h incubation was at 4 °C.

Further studies were performed to assess potential efflux of the Ru(II) complexes in cells. A549 and CHO cells were incubated with Ru-bqp-ester, Ru-bqp-MPP, and Ru-bqp-R8 for 3 h and HPAC cells for 4 h prior to confocal imaging. DRAQ7 was added immediately before imaging to reveal damaged/dead cells.

Co-localization studies were carried out to determine the localization of the complexes at 30 μM. MitoTracker Deep Red (100 nM) was used to selectively stain mitochondria and assess co-localization with the Ru(II) peptide conjugates. MitoTracker Deep Red was selected as a co-localization dye as Ru-bqp-MPP was expected to target the mitochondria and was excited at 644 nm with emission collected between 730 and 820 nm. Pearson’s co-efficient values were calculated using ImageJ software. Briefly, co-localization studies of Ru-bqp-MPP were also completed with LysoTracker Green DND-26 (50 nM) and Rab-7a-GFP, late endosomal stain. We selected just one of the Ru(II) complexes for further co-localization analysis due to the similarity in uptake of the three complexes, with punctate appearance and no uptake in the nucleus across all test cell lines. LysoTracker Green DND-26 was excited at 504 nm and emission collected between 500 and 540 nm and Rab7a-GFP was excited at 488 nm and emission collected between 500 and 540 nm.

### Confocal Imaging of HPAC Spheroids

HPAC cells were seeded in U-bottom 96 well-plates pre-treated with 0.5% polyHEMA [poly(2-hydroxyethyl methacrylate), Sigma in 95% ethanol] at 2 × 10^4^ cells per well. The plate was then centrifuged at 700 rpm at 37 °C using a BMG Labtech ClarioStar (plus) plate reader for 10 min. The cells were incubated for 96 h to allow spheroid formation before any dye was added for imaging studies. Ru-bqp-ester, Ru-bqp-MPP, and Ru-bqp-R8 were added to the spheroids at 30 and 100 μM in the 96 well plates and after 24 h incubation, the spheroids were carefully transferred to an 8-chamber slide (ibidi), with a single spheroid per chamber, and directly imaged using a Leica TCS DMi8 confocal microscope (40× oil immersion objective). Hoechst 33342 nuclear stain (1 μg/mL) was added to the spheroids for 45 min as a contrast agent and excited using a 405 nm laser with emission collected between 425 and 475 nm. The Ru(II) complexes were excited using a white light laser at 490 nm and emission collected between 580 and 730 nm. Spheroid images were acquired using z-scanning across the *z*-axis of the samples. On average, 40–50 images were acquired per z-scan and used to obtain 3D spheroid reconstructions using Leica Application Suite X (LAS X) software.

### Evaluation of Spheroid Viability

Spheroid viability was evaluated using a CellTiter-Glo 3D assay. Although the alamar blue assay is a robust and reliable option for 2D cell viability, the CellTiter-Glo 3D assay is specifically designed to penetrate deeper into 3D cell culture and thus offers better specificity for these studies. Cells were seeded in round-bottom 96 well plates at 2 × 10^4^ cells per well and grown for 96 h. The cells were treated for 24 h and a media change performed. A 470 nm LED was used to irradiate the spheroids at 4.27 ± 0.41 J/cm^2^ (30 min at 2.37 ± 0.23 mW/cm^2^) and a dark control plate was performed simultaneously. Irradiation conditions were tested on untreated spheroids to confirm whether viability remained unaffected at this irradiation dosage before any experiments were performed. The spheroids were allowed to recover and the CellTiter-Glo 3D assay was performed to determine cell viability as per the manufacturer’s protocol. Percentage cell viability was determined relative to the untreated control spheroids.

## Results and Discussion

### Synthesis and Characterization of the Ru(II) Polypyridyl Complexes and Conjugates

**Figure 2:**
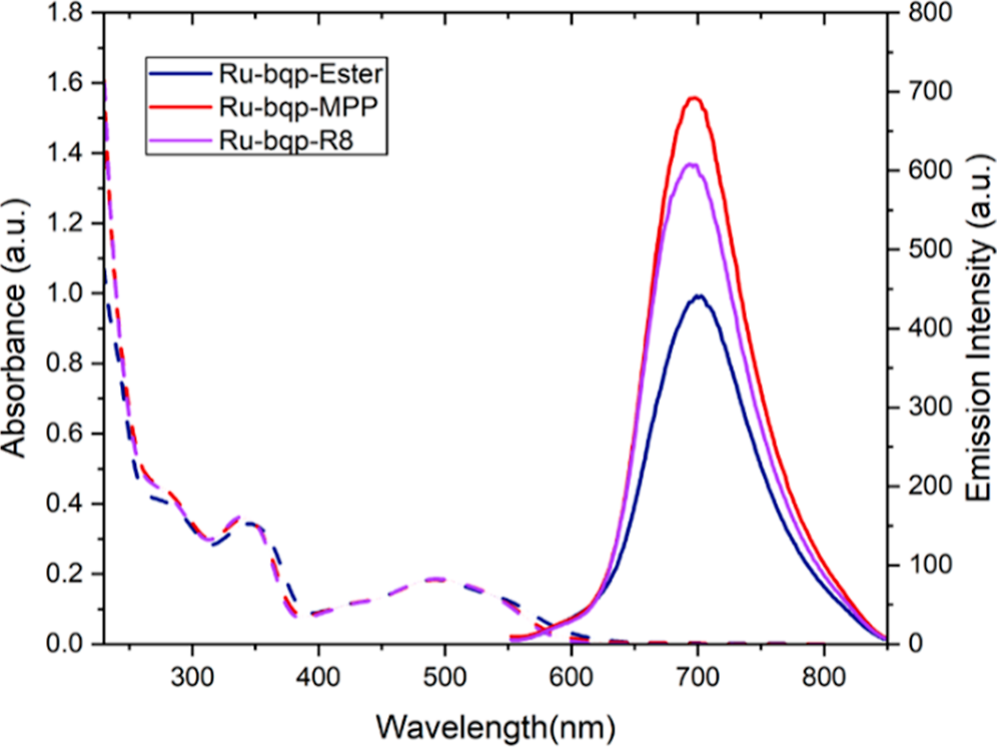
Absorbance and emission spectra of Ru-bqp-ester, Ru-bqp-MPP, and Ru-bqp-R8 (20 μM) in aerated PBS (pH 7.4), emission spectra were excited under absorbance matched conditions at 490 nm so emission intensity directly reflects relative quantum yield changes on peptide conjugation.

Ru-bqp-ester was synthesized by modification of a protocol reported by Abrahammson et al. and the asymmetric complex; [Ru(bqp)(bqp-COOH)](PF_6_)_2_, (Ru-bqp-acid) was prepared to allow peptide conjugation through the carboxy handle. The materials were characterized by ^1^H NMR (Supporting Information. Figures S1–S7) and HR-MS (Supporting Information, Figures S8–S10) and conformed to the previously reported data.^34,57,58^ Peptide conjugation to form Ru-bqp-MPP and Ru-bqp-R8 (Figure 2) were prepared according to methods previously reported by us and also confirmed by H NMR (Supporting Information, Figures S1–S7) and HR-MS (Supporting Information, Figures S8–S10).

### Photophysical Characterization of Ru-bqp-ester, Ru-bqp-MPP, and Ru-bqp-R8

The absorbance and emission of the ester in acetonitrile conform to the previously reported data.^57,58^ The absorbance and emission spectra of the parent, Ru-bqp-ester and peptide conjugates Ru-bqp-MPP and Ru-bqp-R8 in PBS buffer are shown in Figure 2. Emission decay across both ester and the conjugates conformed to single exponential kinetics in PBS and in acetonitrile. In phosphate-buffered saline, the ester complex Ru-bqp-ester, shows long-lived emission of 782 ± 18 ns, which is nearly twice that of the complex in MeCN and the lifetime doubles again to over 1.3 μs on deaeration. This is consistent with data reported previously for related complexes in DI water.^61^ Peptide conjugation had no measurable impact on the optical properties of the complex with absorbance maximum, attributed to MLCT centered around 490 nm in PBS and λ_max_ emission around 690 nm (^3^MLCT). However, emission quantum yield and emission decay increased considerably on peptide conjugation and the magnitude of increase varied with the peptide to 930 ± 40 for R8 and to 1077 ± 33 ns for the MPP conjugate. Similar though more modest effects have been noted previously for ruthenium(II) polypyridyl complexes on peptide conjugation and likely reflects the strong oxygen sensitivity of these species and some limited oxygen protection exerted by the peptide.^62^

To be effective as a type-II sensitizer, one would expect the emission lifetime to be strongly influenced by molecular oxygen and indeed on deaeration in PBS buffer (pH 7.4), emission lifetimes approximately doubled, as listed in Table 1, extending to over 2 μs for Ru-bqp-MPP.

**Table 1:** Photophysical Data for Ru-bqp-ester, Ru-bqp-MPP, and Ru-bqp-R8 in PBS (pH 7.4) and Acetonitrile

	solvent	λ_abs_ (ε) nm (×10^3^ M^–1^cm^–1^)	λ_em_ nm	τ_lum_ ns	
				aerated	deaerated
Ru-bqp-ester	PBS	272 (20.1), 345 (17.8), 491 (9.6)	699	782 ± 18	1320 ± 20
	MeCN	278 (25.6), 344 (23.3), 492 (11.8)	691 (Φ 4%)^a^	384 ± 1	4300^a^
Ru-bqp-R8	PBS	280 (32.0), 342 (30.3), 494 (14.1)	691 (Φ 2.7%)	930 ± 40	1921 ± 49
Ru-bqp-MPP	PBS	278 (37.6), 342 (31.3), 493 (16.0)	690 (Φ 2.2%)	1077 ± 33	2011 ± 21

a Previously published data, measured at 298 K, in a deaerated MeOH/EtOH solution.^48,49^

### Uptake and Distribution in Cellular Monolayers

The uptakes of Ru-bqp-ester, Ru-bqp-MPP, and Ru-bqp-R8 at 37 °C were assessed across three mammalian cell lines, two cancer; A549 and HPAC and one non-cancer; CHO, by incubating the complex or conjugates at 30 μM in phenol red free media. All three Ru(II) complexes including rather expectedly, the parent complex, Ru-bqp-ester, exhibited good membrane permeability and cellular uptake at live cells. Indeed, all showed notably rapid permeation, particularly at CHO and A549 cells where the uptake was completed by 30 min. Consequently, for cellular imaging, CHO and A549 cells incubation time of just 1 h was applied, while HPAC cells required a minimum incubation of 2 h to achieve a complete uptake, with images acquired at this cell line after 4 h incubation (Figure 4).

**Figure 3:**
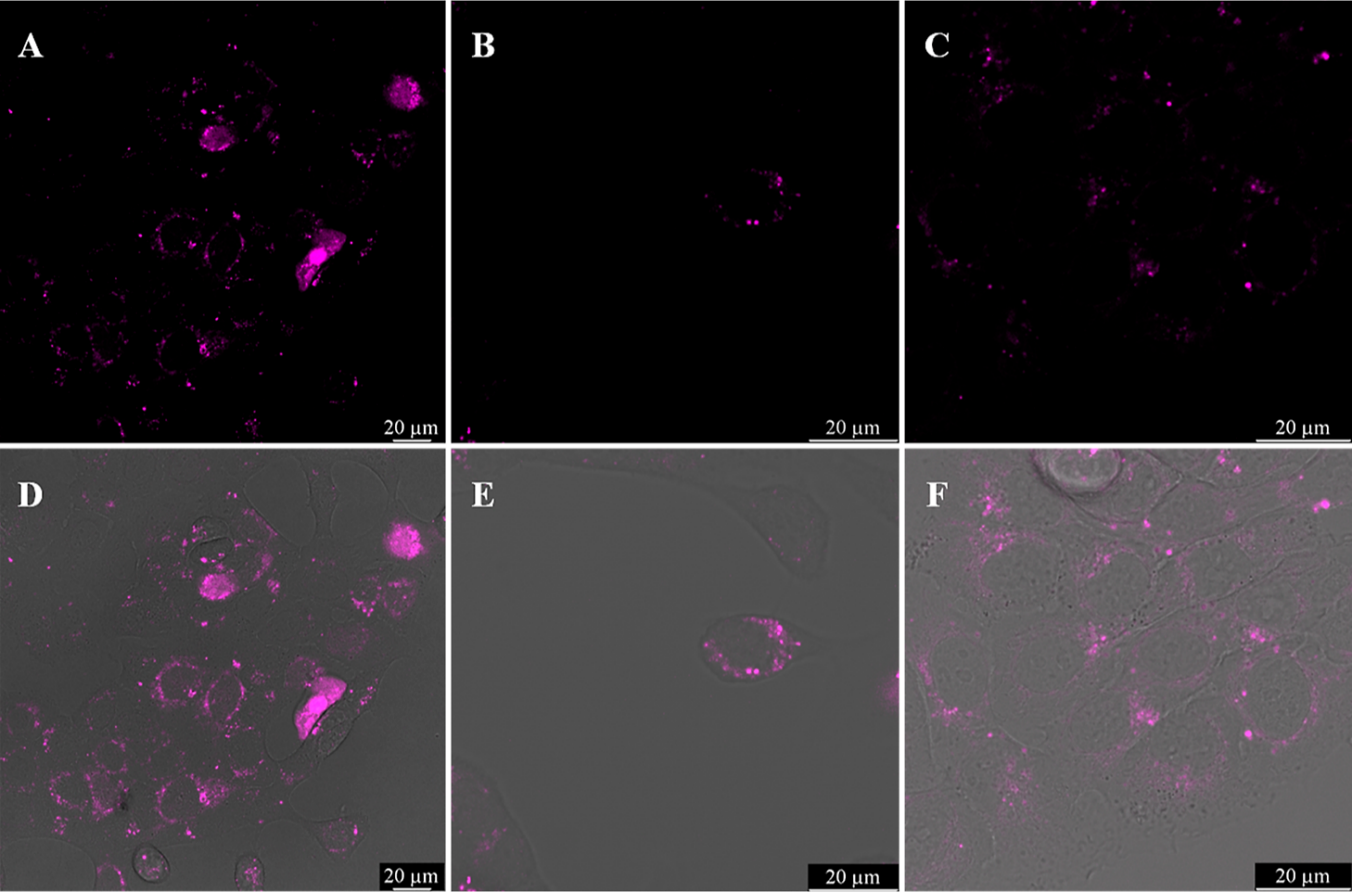
Cellular uptake of 30 μM (A) Ru-bqp-MPP, (B) Ru-bqp-R8, and (C) Ru-bqp-ester in HPAC cell monolayers at 37 °C and 4 h incubation, with their (D,E) corresponding brightfield overlay images. 470 nm from a white light laser was used to excite the Ru (II) peptide conjugate and emission was collected between 580 and 700.

**Figure 4:**
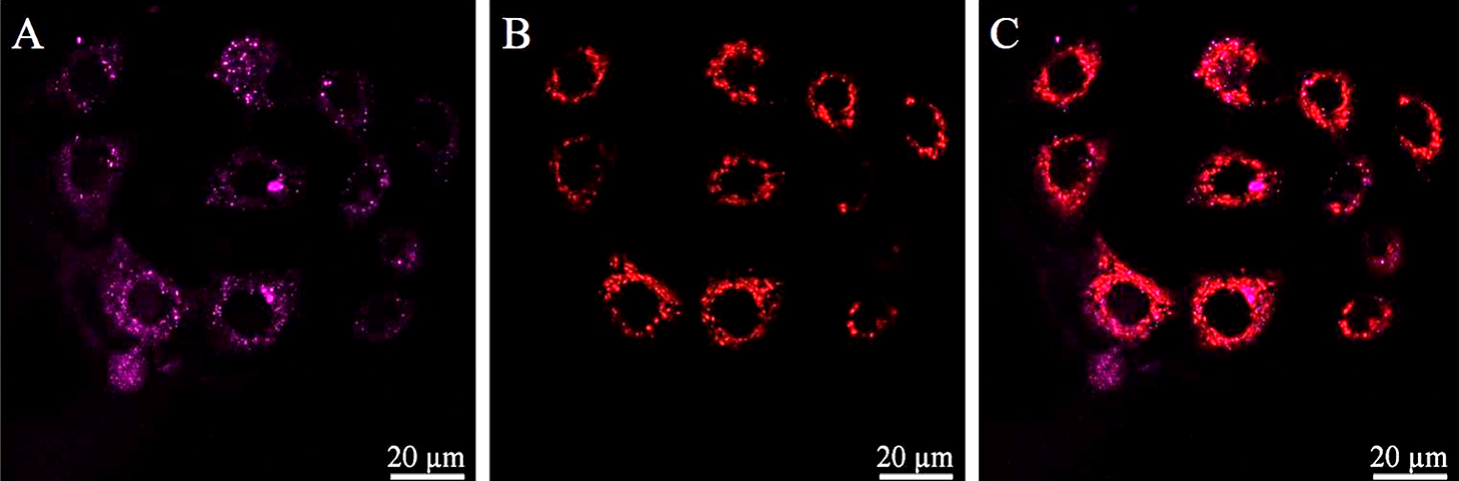
Confocal luminescence imaging from the cellular uptake and localization studies of Ru-bqp-MPP in live A549 cells. Cells were incubated for 1 h in the dark with (A) Ru-bqp-MPP and co-stained with (B) MitoTracker Deep Red (100 nM) for 30 min. Mitochondrial penetration is evident but cCo-localization was relatively low, confirmed in (C) the overlay image of Ru-bqp-MPP and MitoTracker Deep Red (Pearson’s coefficient = 0.302 ± 0.05). 470 nm from a white light laser was used to excite the Ru (II) peptide conjugate and emission was collected between 580 and 700 nm. The MitoTracker Deep Red dye was excited at 644 nm and emission collected between 730 and 820 nm.

After the uptake, the probe distribution in live cells is remarkably similar across all three complexes, irrespective of peptide conjugation and cell type. In all cases, emission was evident throughout the cytoplasm with intense punctate features overlying weaker continuous emission. All three complexes appear to be nuclear excluding, but the emission is particularly bright around the exterior of the nucleus.

Given the notable speed of uptake, temperature-dependent uptake studies were carried out in CHO and A549 cells at 4 °C to determine the mechanism of uptake of the Ru(II) complexes. Most reported metal complex peptide conjugates, certainly all reported to date by our group, undergo cell uptake by endocytosis or active transport, where the uptake was observed to switch off at 4 °C, indicating an energy-dependent pathway.^23,29,63,64^ By contrast here, all three compounds remained strongly cell permeable at 4 °C and imaging following 1 h incubation confirmed, uniquely in our experience, that the mechanism of uptake is energy independent. This accounts for the rapid uptake by live cells. The absence of energy requirement/passive uptake also explains the membrane permeability of the parent Ru-bqp-ester complex and it is reasonable, though surprising, that the peptide conjugates derived from this complex exhibited the same mode of uptake. Generally, ruthenium complexes without peptide conjugation have poor membrane permeability in the absence of permeabilization agents, such as detergent or DMSO. The parent and peptide conjugates were relatively water soluble, requiring negligible DMSO to prepare the 2 mM starting stock solutions, where overall a minimal final DMSO concentration of <0.003% V/V would have been present in the cell incubation medium.

Interestingly, and also not observed previously in related peptide conjugates, confocal imaging studies of A549 cells indicate efflux of the complexes may be occurring. Cells were incubated as usual at 37 °C in the dark and washed twice with PBS prior to imaging. The wash step serves to both remove any of the complex sitting on top of the cells but also to remove any dead cells. In live A549 cells, after peaking around 1 h incubation, emission intensity diminished over time and, after a 3 h incubation, no emission could be observed under our routine imaging conditions. However, as shown in Figure S13, using Ru-bqp-MPP as an example, the complex was evident in dead A549 cells, as confirmed by the DRAQ7 uptake. This behavior was not observed in CHO and HPAC cell monolayers, where molecular brightness remained unchanged beyond 3 and 4 h incubation, respectively. Further investigation is necessary, but our preliminary data suggest that efflux is occurring from this cell line.

To investigate the distribution of the complexes in-cellulo further, co-localization studies with MitoTracker Deep Red were carried out in A549 cells and revealed a surprisingly low correlation between the emission of the Ru-bqp-MPP complex and MitoTracker Deep Red (Figure 3) with a Pearson’s coefficient of 0.302 ± 0.05, while for Ru-bqp-R8, a Pearson’s coefficient of just 0.163 ± 0.02 was determined. The study indicates that although Ru-bqp-MPP does not show exclusive targeting to the mitochondria, as previously observed for complex conjugates carrying this mitochondrial targeting sequence,^45,62^ it does penetrate the mitochondria compared to the general cell permeable peptide (CPP), R8. Although poor specific targeting of the Ru-bqp-MPP conjugate was surprising, we have seen less predictable targeting with MPP or nuclear localizing signal (NLS) peptides previously when combined with highly lipophilic Ru complexes, such as biquinoline or diphenyl phenanthroline [Ru(biq)_2_(trz-CONH-MPP)]^4+^ and [Ru(biq)_2_(trz-CONH-NLS)]^5+^ studied in CHO and HeLa cell lines.^65,66^ Similarly, co-localization of Ru-bqp-MPP with LysoTracker Green DND-26 in A549 and Rab7a-GFP in A549 and CHO cells, indicated Pearson’s values of 0.197 with LysoTracker Green DND-26 and 0.117 and 0.105 for Rab7a-GFP in A549 and CHO cells, respectively (Figure S16). This observation and the evidently wide distribution of the three Ru(II) complexes in the cytoplasm along with their rapid uptake by passive diffusion indicates mainly cytoplasmic staining. A similar distribution of the Ru(II) complex and conjugate was observed across all CHO, A549, and HPAC cell monolayers, with no uptake evident in the nucleus of the cells.

### Dark Toxicity in Cell Monolayers

The cytotoxicity of Ru-bqp-ester and its peptide conjugates, Ru-bqp-R8 and Ru-bqp-MPP is an important consideration in assessing their suitability as imaging or as potential PDT agents, where for both applications dark toxicity should be low. The alamar blue (Resazurin) assay was used to assess cell viability of CHO, A549, and HPAC cell monolayers after 24 h incubation in the dark with the complexes and conjugates. In the presence of metabolically active cells, the alamar blue dye is reduced to resorufin and changes from navy blue to a pink color depending on the number of viable cells. Thus, this assay can be used as a simple direct indicator of cell viability. CHO and A549 cells were treated with the peptide conjugates at concentrations of 0, 1, 5, 10, 15, 25, 50, 75, and 100 μM and HPAC with concentrations of 0, 15, 25, 50, 75, 100, and 150 μM. In the case of Ru-bqp-ester additional concentrations (200 μM, 500 μM, and 1 mM) were included in the assay to obtain an IC_50_ value (Figure S17).

The parent complex, Ru-bqp-ester, was minimally toxic toward all the cell lines assessed, with an IC_50_ value above 100 μM in all cases when incubated for 24 h in the dark. Similarly, in A549 cells, the peptide conjugates showed low dark toxicity, with an IC_50_ value above 100 μM. By comparison, after 24 h incubation, the R8 and MPP peptide conjugates showed modest dark toxicity toward CHO and HPAC cell lines with IC_50_ of 50 and 20.56 ± 1.89 μM for MPP and 78 and 42.66 ± 2.52 μM for R8 in CHO and HPAC cells, respectively. It is important to note that the IC_50_ values obtained for our dark control plates and 24 h dark toxicity studies will vary as the recovery time for the dark control plate is significantly longer than for the cytotoxicity studies. Cells in our dark control plates are incubated with Ru(II) for 30 min and allowed 23.5 h to recover prior to the alamar blue assay, whereas cytotoxicity studies involve treating the cells for 24 h, and adding the 10% (v/v) alamar blue in a media solution immediately after the incubation, giving the cells no time to recover. Our findings confirm that cell death is triggered by both the MPP and R8 conjugates in a dose-dependent manner that enabled us to identify a safe working concentration for imaging and phototoxicity.

### Phototoxicity

**Figure 5:**
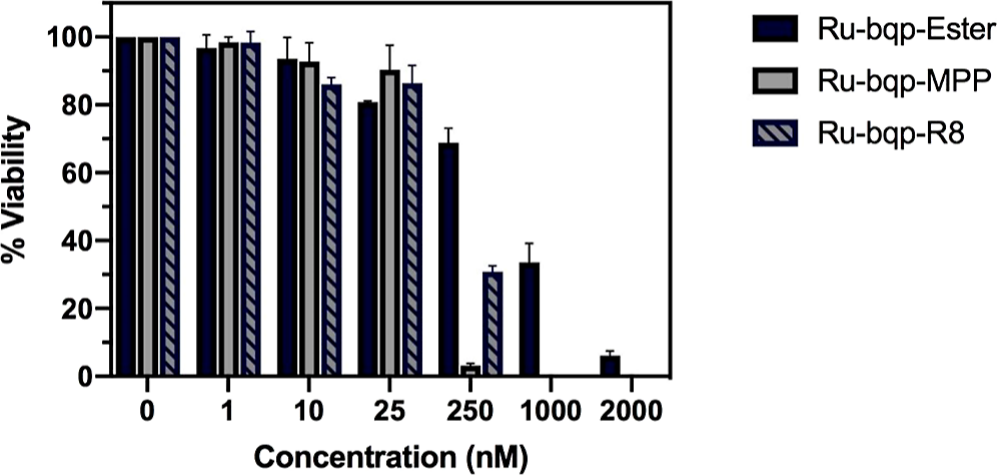
Cell viability of HPAC cells after 2 h exposure to Ru-bqp-ester, Ru-bqp-MPP, and Ru-bqp-R8 at 0, 1, 10, 25, 250, 1000, and 2000 nM and irradiation with a 470 nm blue light LED to a final irradiation dose of 17 J/cm^2^ ± 1.64 J/cm^2^ (2 h at 2.37 ± 0.23 mW/cm^2^).

The phototoxicities of the Ru-bqp-ester, Ru-bqp-R8, and Ru-bqp-MPP were next compared across the three cell line monolayers. Two identical plates, a dark control and phototoxicity plate, were prepared and incubated with varying concentrations of Ru-bqp-ester, Ru-bqp-R8, and Ru-bqp-MPP for 30 min or 2 h depending on the cell line. The test solutions were then decanted, and the wells replaced with phenol red free media. The phototoxicity plate was irradiated using a 470 nm LED, corresponding to which is close to the absorbance maximum associated with the ^1^MLCT of the complex, total irradiation dose 17 ± 1.64 J/cm^2^ (constituting 2 h at 2.37 ± 0.23 mW/cm^2^) at 37 °C, and the control plate was maintained under the same conditions in the dark. The cells were then incubated for a further 21.5 h at 37 °C with 5% CO_2_ to allow for cell recovery. An alamar blue assay was performed to assess cell viability. For phototoxicity studies, we selected an irradiation dose close to clinically used ranges, which are typically between 20 and 200 J/cm^2^ as possible, which also maintained cell viability.^67^Figure 5 shows a bar chart of the % cell viability for HPAC cells and the same plots for CHO and A549 are shown in the Supporting Information.

The PI values are provided in Table 2. To overcome potential efflux of the complexes from the A549 cells, a shorter complex incubation time of 30 min was selected prior to irradiation. This was confirmed from imaging to be sufficient incubation period to allow for cell uptake, without loss of emission intensity. In contrast to the dark toxicity controls, cell viability was extremely low in A549 cells following photoirradiation. More than 70% of A549 cells were dead when treated with the parent complex Ru-bqp-ester or the Ru-bqp-MPP and Ru-bqp-R8 peptide conjugates at a dose of <5 μM, i.e., IC_50_ under irradiation was below 5 μM.

**Table 2:** Cytotoxicity and Phototoxicity Half-Maximal Inhibitory Concentration (IC_50_) Values (μM) in HPAC Cell Monolayers and Multicellular Tumor Spheroids^a^

	HPAC 2D cell monolayers			HPAC multicellular spheroids (3D)		
	dark (μM)	light (μM)	PI^b^	dark (μM)	light (μM)	PI^b^
Ru-bqp-ester	585 ± 6.28	1.77 ± 0.11	760	>100	>100	
Ru-bqp-MPP	27.39 ± 1.06	0.047 ± 0.01	582	>100	30.29 ± 3.17	>3.3
Ru-bqp-R8	64.78 ± 0.55	0.104 ± 0.03	623	>100	41.50 ± 4.12	>2.5

a IC_50_ values in μM, toxicity evaluated from the average of three plates (cells treated in triplicate per plate).

b PI = phototoxic index, the ratio of dark to light IC_50_ values. Cell monolayers irradiated at 17 ± 1.64 J/cm^2^ and spheroids at 4.27 ± 0.41 J/cm^2^ (irradiation dose selected as appropriate for cells or spheroids without inducing toxicity for non-treated control samples).

Similar to the phototoxicity studies in A549 cells, an incubation time of 30 min was selected for CHO cells to allow uptake. As the parent complex, Ru-bqp-ester was not toxic toward CHO in the dark, we were interested to assess the phototoxicity of the complex toward this non-cancerous cell line. Similar to the A549 and HPAC cells, the Ru-bqp-ester complex was found to be phototoxic in CHO with an IC_50_ of below 5 μM. Cell viability was determined relative to untreated control wells using an alamar blue assay. All plates were performed in triplicate (S18).

The HPAC cells required a longer incubation period of 2 h prior to irradiation to account for slower uptake. Comparing the Ru(II) parent ester complex and its peptide conjugates in HPAC cells (Figure 5) an IC_50_ under irradiation of just 770 ± 110, 47 ± 12, and 104 ± 30 nM for Ru-bqp-ester, R8, and MPP, respectively, was determined. Therefore, all compounds showed impressive phototoxic index (PI) values from Ru-bqp-MPP at 582, Ru-bqp-R8 at 623, and Ru-bqp-ester at 623 (Table 2). It is important to note that impressive PI values for other ruthenium polypyridyl complexes have been reported recently, most notably a PI value of 5439 in HL-60 cells; however, in reported cases, higher irradiation doses, e.g., 28 J/cm^2^ to 100 J cm/cm^2^, was administered in these studies.^68−71^ In summary, Ru-bqp-ester is minimally toxic in the dark but highly phototoxic in all test cells when activated using a 470 nm LED. Furthermore, the peptide conjugates Ru-bqp-R8 and Ru-bqp-MPP show phototoxic potential in cancerous cell lines A549 and HPAC selected for analysis. The increased uptake of the MPP peptide conjugate into the mitochondria, as determined by our co-localization studies, could be linked to the higher phototoxicity of the complex in A549 and HPAC cells and also the increased dark toxicity in HPAC and CHO cell monolayers in comparison to the parent and R8 peptide conjugate.

It is important to highlight that a direct comparison between the other reported PIs is difficult to make because of the diversity of conditions used to determine PI values. Because PI is influenced by a range of factors, including the cell type extent of permeability of a given agent and the irradiation dose.^72^ Nonetheless, our PI values seem to be among the highest reported for Ru(II) polypyridyl complexes and exceed many reported PI values of clinically approved PS under comparable conditions.^73−75^ Based on control studies with untreated CHO, A549, and HPAC cell monolayers, we selected an irradiation dose of 17 ± 1.64 J/cm^2^ because this intensity was observed in the control not to impact the cells directly and because similar irradiation conditions have been used by others, for example, Cloonan et al. applied 18 J/cm^2^ to study the phototoxicity of their photoactive Ru(II) complexes.^76^ For approved PDT agents, experimental conditions used for reported PI vary for example, the phototoxicity of Photofrin has been extensively studied, with the reported PI values of at least 10, though the irradiation dose is often lower than those used here. For example, PI of Photofrin was reported to be >10 in HeLa cells with an irradiation dose of 5 J/cm^2^ by Delaey et al.^77^ Additionally, PI values of >18 and 4.2 were reported for clinically approved PDT drug aminolevulinic acid (ALA) by Glazer and colleagues in HL60 leukemia cells and A549 cell monolayers, respectively.^78^

The thiophene pendants in TLD-1433 are reported to serve to delocalize the excited state of the complex reducing its energy and promoting its triplet ligand character and lifetime to promoting triplet energy transfer to oxygen.^79^ As described the expansion of the coordination cage in complexes like Ru-bqp can similarly extend triplet lifetime and promote triplet energy transfer. Thus, it is anticipated that the origin of phototoxicity is ^1^O_2_ or ROS generation. To identify singlet oxygen production, a ^1^O_2_ scavenger study was completed using 1,3-diphenylisobenzofuran (DPBF). In the presence of ^1^O_2_, DPBF forms endoperoxide 1, which decomposes to the colorless 1,2-dibenzoylbenzene (DBB) that can be detected spectrophotometrically through decreases to the DPBF absorbance at 410 nm. Although highly sensitive to ^1^O_2_, it is important to note that DPBF can also react with other ROS, for example, O_2_^–^ or hydroxyl radicals.

A solution of 10 mM DPBF containing 20 μM of the parent complex Ru-bqp-ester in ethanol (with 1% DMSO) was assessed for ^1^O_2_ generation by photoirradiating the sample with a 470 nm LED at 2.37 ± 0.23 mW/cm^2^. Within 5 min of irradiation of the DPBF-Ru solution was degraded with a decrease in an absorbance of 77 ± 4% (Table S1), indicating the phototoxicity of the Ru(II) complexes is the result of ^1^O_2_ generation. A control study was performed by irradiating the DPBF without the Ru-bqp-ester to confirm that direct photodecomposition of the DPBF was <10% decrease in absorbance, which was observed over *t* = 0 and *t* = 6 min of irradiation.

### 3D Cell Culture—Multicellular Tumor Spheroids

While imaging and cytotoxicity of metal complex luminophores have been widely investigated in the 2D culture, they have been far less studied in multicellular structures. And, particularly in the context of PDT, very little evaluation has been extended to the study of metal complex peptide conjugates in 3D cell models. However, spheroids can provide a more physiologically realistic insight into the potential effectiveness of PDT reagents in vivo. We thus evaluated the uptake, toxicity, and photocytotoxicity of the parent and conjugates in HPAC MCTS.

**Figure 6:**
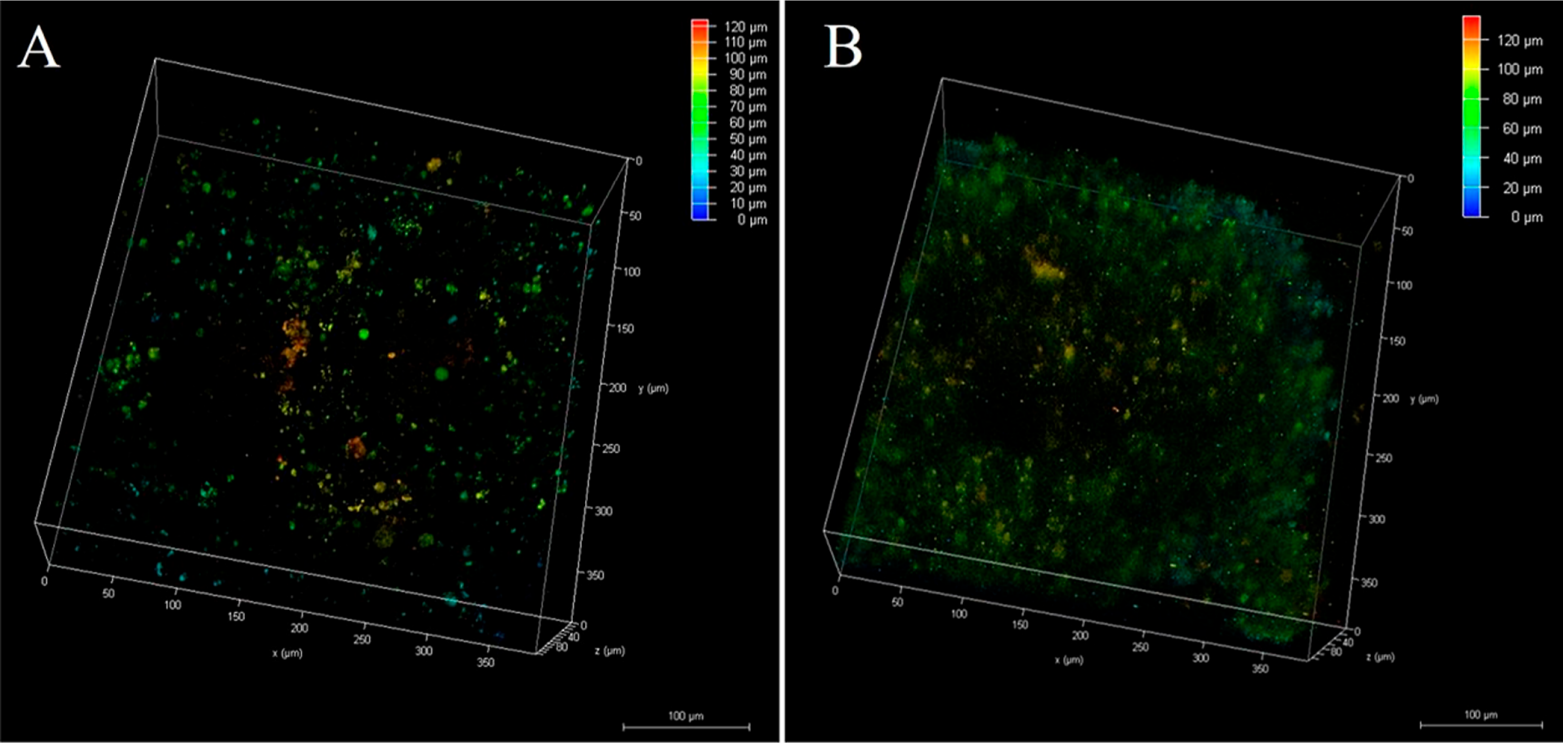
3D reconstruction depth coding images of live HPAC spheroids with Ru-bqp-ester at (A) 30 and (B) 100 μM at 24 h incubation in the dark obtained by collecting images in a z-scan from below to above the spheroid. 490 nm from a white light laser was used to excite the spheroids with Ru(II), and emission was collected between 580 and 730 nm.

**Figure 7:**
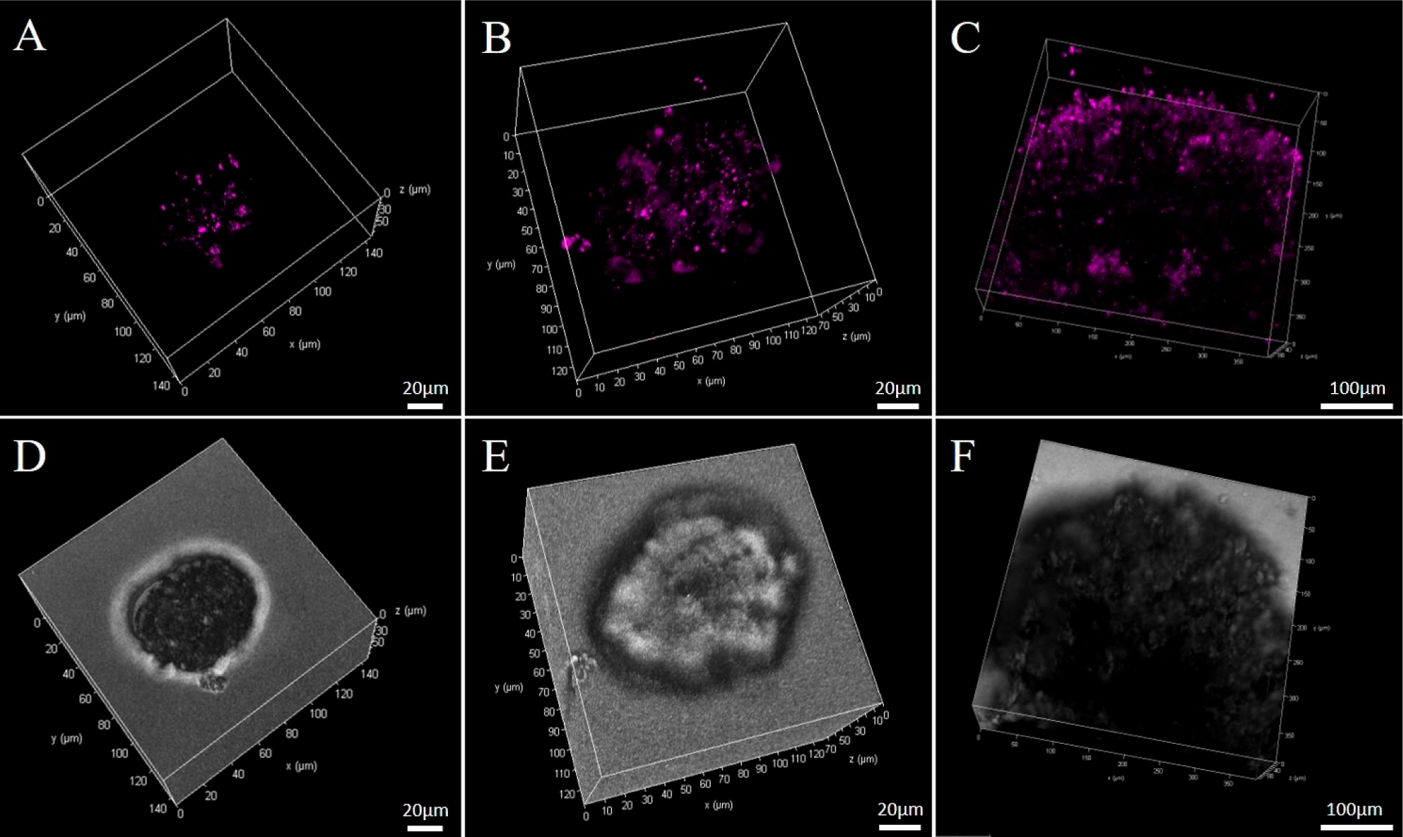
3D reconstruction images of live HPAC spheroids treated with (A) Ru-bqp-ester, (B) Ru-bqp-MPP, and (C) Ru-bqp-R8 at 100 μM for 24 h and (D,E) the brightfield images of the respective spheroids. 490 nm from a white light laser was used to excite the spheroids and emission was collected between 580 and 730 nm.

Confocal fluorescence imaging was performed to determine if the cellular uptake observed in 2D culture extends to 3D models. The HPAC MCTS were grown for 96 h, treated with Ru-bqp-ester, Ru-bqp-MPP, or Ru-bqp-R8 for 24 h at 30 and 100 μM before imaging. A 24 h incubation window was selected to mimic the majority of current PDT protocols, which involve the administration of a PS approximately 24–48 h prior to surgery.^80^ For example, Raza and colleagues studied the uptake of a ruthenium (II) complex [{Ru(TAP_2_)}_2_(tpphz)]^4+^ in 3D melanoma spheroids prepared using the C8161 cell line and confirmed in that case that a 24 h incubation period of the complex at 100 μM was sufficient to ensure uptake.^81^ Confocal imaging of HPAC spheroids with Ru-bqp-ester, Ru-bqp-MPP, and Ru-bqp-R8 confirmed some uptake at 30 μM, the concentration used for imaging of 2D culture. However, a higher concentration of 100 μM ensured complete internalization of the complex into the 3D structure. This was confirmed from depth coding images of Ru-bqp-ester incubated with 30 and 100 μM for 24 h prior, as shown in Figure 6, where a clear contrast in the accumulation of ruthenium at 30 μM (Figure 6A) and 100 μM (Figure 6B) can be seen. As for the 2D studies, the spheroid studies indicate a similar cellular uptake between the parent and peptide conjugates in the spheroids. As shown in Figure 7, there is a wide distribution of the complexes throughout the spheroids, confirming they are capable of penetrating deeply throughout the spheroid, with no evidence of sub-cellular localization. Overall, the data indicates both the parent and peptide conjugates can be delivered effectively to cells in a multicellular environment and thus shows potential for use as therapeutic agents.

### Toxicity in a 3D Cell Model (Spheroids)

The dark and phototoxicity of the parent and peptide conjugates were also assessed in HPAC spheroids. Control studies were performed with untreated spheroids to determine the appropriate irradiation dose for phototoxicity studies. The spheroids were treated with 0, 5, 25, 50, and 100 μM of the parent and peptide conjugates for 24 h, fresh phenol red free media was added, and the plate was irradiated with a 470 nm LED at a total dose of 4.27 ± 0.41 J/cm^2^ (30 min at 2.37 ± 0.23 mW/cm^2^). The spheroids were incubated for a further 23.5 h in the dark to allow for recovery. Cell viability was determined using the CellTiter-Glo 3D luminescence-based assay, and viability assays were performed in triplicate. The CellTiter-Glo 3D reagent can penetrate deeply into 3D cell cultures and detect the presence of ATP, an indicator of cell viability. As in the 2D alamar blue assay protocol, % viability is calculated relative to untreated control spheroids.

Phototoxicity assays performed with HPAC spheroids confirmed that the phototoxicity of the ruthenium complexes observed in 2D cell monolayer studies persists in 3D spheroids. As previously mentioned, 3D cell culture models closely mimic the in vivo environment of tumors compared to experiments using cell monolayers. Therefore, there is often a difference in uptake, distribution, and phototoxic effects in 3D versus 2D cell cultures. In comparison to the phototoxicity studies performed in HPAC cell monolayers, where the cells required only a 2 h incubation with the ruthenium complexes, the spheroids were treated for 24 h prior to irradiation. As outlined in Table 2, the PIs of Ru-bqp-ester, Ru-bqp-MPP, and Ru-bqp-R8 were lower when tested in 2D compared to 3D HPAC cell models. While the dark IC_50_ values of the complexes were above 100 μM, indicating low dark toxicity, we confirmed that both the peptide conjugates Ru-bqp-MPP and Ru-bqp-R8 had PIs of above 2. As per the OECD TG 432,^82^ a PI value between 2 and 5 indicates probable phototoxicity and a PI value above 5 suggests positive phototoxicity; thus, as the PI values achieved for Ru-bqp-MPP and Ru-bqp-R8 were in the >2 and <5 categories for our 3D toxicity studies in HPAC spheroids, the phototherapeutic potential of the conjugates carries across 2D to 3D studies.

The notably lower phototoxicity of the Ru-bqp-ester complex toward 3D cell models compared to the peptide conjugates was surprising given their relatively similar effects in the 2D-cell culture. This we tentatively attribute to potentially improved penetration of Ru-bqp-MPP and Ru-bqp-R8, leading to their potentially higher accumulation into vulnerable organelles, particularly mitochondria in the spheroid compared to the ester. Although the uptake on the basis of intensity seemed to be only modestly better in the case of the peptides from confocal imaging, the localization of Ru-bqp-ester, Ru-bqp-MPP, and Ru-bqp-R8 was not distinguished in imaging of the HPAC spheroids, but given the improved mitochondrial penetration was observed, particularly in the case of Ru-bqp-MPP in the 2-D culture, this is a rational conclusion.

It is important to note that although there was a significant decrease in phototoxicity in spheroids with an IC_50_ of >100 μM for Ru-bqp-ester, 30.29 ± 3.17 μM for Ru-bqp-MPP, and 41.50 ± 4.12 μM for Ru-bqp-R8, this can in large part be ascribed to the reduced irradiation dose the spheroids received in comparison to the cell monolayers. We found that a lower irradiation dose was necessary from control studies performed on the spheroids in media, without sensitizers, that indicated an irradiation dose of 17 ± 1.64 J/cm^2^ used for cell monolayer studies had a direct toxic impact on the spheroids. Therefore, the irradiation time and consequently the total irradiation dose for 3D toxicity studies was reduced to a level that showed no toxicity in the controls, which is about 4× lower than that applied to 2D culture. This does not fully account for the difference observed though. While cell monolayers can quickly repair the damage caused by the LED/sensitizer, spheroids have a reduced capacity for repair because their compact nature reduces oxygen availability and nutrients in the inner layers that along with their slower proliferation rate reduces their recovery from radiation damage compared to cell monolayers. In addition, the inner layers of spheroids, may be shielded somewhat from the irradiation, as scatter will reduce the penetrative power of the light and therefore, the ruthenium that is accumulating in this region may not being excited as efficiently by the LED. The oxygen concentration is also expected to be depleted at the spheroid core, depending on the spheroid size, and assuming based on the singlet oxygen studies above that it is a type II PDT agent, the reduced molecular oxygen at the core will reduce the effectiveness of the Ru(II) complexes if operating through a type-II mechanism. Of note, however, typically spheroids of larger than 200 μm begin to show a hypoxic center and require a minimum of 4 days to form the necrotic zone depending on the cell line used, with 500 μm being the optimum spheroid size for the formation of necrotic zones.^83,84^ Our spheroids were grown for 4 days, to an diameter of 100 to 250 μm, thus while our spheroids may not have a hypoxic core like larger spheroids, they will still have less oxygen at their core due to their size. Although the complex and conjugates show excellent PI in cell monolayers and spheroid penetrability, the reduced PI at the spheroid reflect better the challenges and performance of the complexes and their phototoxic potential in a tumor environment.

Similar increases in the IC_50_ values and therefore, a decrease in the PIs of phototoxic agents between 2D and 3D cell culture models have been noted before. For example, Kucinska et al. have reported that IC_50_ values of zinc phthalocyanine M2TG3 in spheroids prepared and grown for 3 days using the prostate cancer cell line LNCaP are almost four times higher than the IC_50_ values obtained for 2D cell monolayers of the same cell type under normoxic conditions.^85^ However, in our case, as the irradiation dose selected for the HPAC spheroids is lower than what was tested on the cell monolayers, it may account for the lower PI values obtained. Overall, the contrast in toxicity values obtained in 3D versus 2D cell models highlights the importance of evaluating the efficacy of PS’s in both models to gain a better understanding of their potential for use in PDT.

## Conclusions

A luminescent ruthenium(II) polypyridyl complex Ru-bqp-ester and its peptide conjugates, Ru-bqp-MPP and Ru-bqp-R8, exhibited excellent cell permeability with a rapid energy-independent uptake in HPAC, A549, and CHO cells in growth media. The parent complex was minimally cytotoxic toward A549, CHO, and HPAC cells with IC_50_ values above 100 μM, while the MPP and R8 peptide conjugates showed moderate cytotoxicity toward HPAC and CHO cells. Under conditions used for cellular imaging, cells remained viable, confirmed by the lack of DRAQ7 uptake during the confocal imaging window. However, under continuous photoirradiation using a 470 nm LED, the parent complex and peptide conjugates showed high phototoxicity in both A549 and HPAC cancer cell lines with PI values ranging from 580 to 750 in HPAC cells. The assessment by ^1^O_2_ scavenger, DPBF, indicated that the mechanism is type II, as expected. Interestingly, conjugating the parent complex to a cell penetrating octaarginine sequence or a mitochondrial targetting sequence results in increased dark cytotoxicity in the CHO and HPAC cell lines. Co-localization studies with MitoTracker Deep Red confirmed mitochondrial penetration, particularly for the MPP conjugate, but showed a low Pearson’s coefficient value, which indicated mainly non-specific localization of Ru-bqp-MPP and R8 within the mitochondria of A549 and CHO cell monolayers. Nonetheless Ru-bqp-MPP was found to be the most toxic of the three Ru(II) complexes toward the test cells used in this study. Although targeting is not specific, the greater toxicity of Ru-bqp-MPP is likely due to superior penetration of this complex to the mitochondria than the other complexes reflected in the higher Pearson’s coefficient with Mitotracker. The cellular uptake and phototoxicity of these complexes observed in the 2D cell culture was shown to extend to 3D culture in human pancreatic MCTS. The parent and peptides exhibited similar localization and deep penetration throughout 3D spheroids. Photocytotoxicity was observed in the HPAC spheroids but was reduced, notwithstanding lower irradiation powers, compared to the 2D cell monolayers. Overall, this work demonstrates that ruthenium complexes with enhanced ligand metal-centered triplet states make potentially valuable nominees as phototherapeutics, for example, as photosensitizers for PDT.

## Supporting Information Available

Synthetic procedures and characterization (including ^1^H NMR and mass spectra); lifetime decays of aerated and de-aerated Ru-bqp-MPP; confocal imaging; toxicity assays; and 3D cell studies (PDF)
