# Supplementary material for: Phototoxicity of Tridentate Ru(II) Polypyridyl Complex with Expanded Bite Angles toward Mammalian Cells and Multicellular Tumor Spheroids
Source: Inorg Chem. 2023 Aug 3;62(32):13089–102. doi: 10.1021/acs.inorgchem.3c01982 (PMC10428208; doi:10.1021/acs.inorgchem.3c01982)
Supplement: Supplementary file 1 — ic3c01982_si_001.pdf [file ic3c01982_si_001.pdf]

Supplementary Information for:

Phototoxicity of Tridentate Ru(II) Polypyridyl Complex with Expanded Bite Angle toward Mammalian Cells and Multicellular Tumor Spheroids.

Rhianne C. Curley[a], Christopher S. Burke[a¥], Karmel S. Gkika[a], Sara Noorani[b], Naomi Walsh[b] and Tia E. Keyes[a]

a School of Chemical Sciences, National Centre for Sensor Research, Dublin City University, Dublin 9, Ireland, D09 NA55
b School of Biotechnology, National Institute for Cellular Biotechnology, Dublin City University, Dublin 9, Ireland, D09 NA55
¥ Present address: School of Chemistry and Analytical and Biological Chemistry Research Facility, University College Cork, Co. Cork, Ireland, T12 K8AF

Corresponding author's email: tia.keyes@dcu.ie

Table of Contents

Synthesis and Structural Characterisation 3
General Information 3
Characterisation Data 5
Photophysical Studies 11
O_2 Sensitivity Studies 11
Cell Studies 12
Confocal Laser Scanning Microscopy (CLSM) 12
Toxicity 14
3D Cell Studies 15

Synthesis and Structural Characterisation

General Information

Synthesis of *mer*-[Ru(bqp)(bqp-COOH)]$(PF_6)_2$ (Ru-bqp-acid)

mer-[Ru(bqp)$(CH_3CN)_3$]$(PF_6)_2$ (250 mg, 0.29 mmol) and bqpCOOEt (132 mg, 0.33 mmol) were heated at reflux in ethylene glycol (15 mL) for 72 hours. The deep-red reaction mixture was cooled and poured on a stirring solution of NH_4PF_6 to precipitate crude [Ru(bqp)(bqp-'ester')]$(PF_6)_2$ which was filtered and washed with water. The Ru-ester material was partially purified by dissolution in acetone (30 mL), filtering out insoluble solids, and evaporation of the red filtrate to provide a dry residue. Next, dried crude [Ru(bqp)(bqp-'ester')]$(PF_6)_2$ was suspended at room temperature in a mixture of 4:1 THF:CH_3OH (50 mL) and was treated with a solution of LiOH.H_2O (126 mg, 3.0 mmol) in 10 mL water. The combined reaction mixture was stirred overnight in the dark. Upon completion, the reaction volume was concentrated and a red solid was isolated by filtration after the addition of NH_4PF_6 in 1 M HCl (aq.) (50 mL). The crude Ru-acid material was purified by column chromatography on silica gel using 90/10/1 $CH_3CN/H_2O/KNO_3$ (20% aq.) as eluent. *mer*-[Ru(bqp)(bqpCOOH)]$(PF_6)_2$ was isolated from the dried deep-red product band by twice precipitating a solid from acetone solutions using NH_4PF_6 in 1 M HCl (aq.). Yield: red solid, 146 mg (45 %). ^1H NMR (600 MHz,CD_3CN) δ (ppm): 7.01 (dd, 2H); 7.06 (dd, 2H); 7.44 (q, 4H); 7.65 (2d, 4H); 7.70 (d, 2H); 7.80 (d, 2H); 7.87 (d, 2H); 8.03 – 8.05 (m, 6H); 8.08 (d, 2H); 8.15 (t, 1H); 8.30 (s, 2H). ^{13}C NMR (CD_3CN) δ (ppm): 122.69, 123.07, 127.52, 127.58, 127.65, 127.91, 128.61, 128.81, 131.06, 131.51, 132.77, 133.32, 133.82, 133.94, 138.37, 138.43, 138.96, 147.49, 147.57, 157.22, 157.81, 159.19, 159.62, 166.48. HR-MS (ESI(+)-qTOF) *m/z*: 812.1470 $[M – 2PF_6^-]^+$; Calculated for $C_{47}H_{30}N_6O_2Ru$: 812.1474.

Synthesis of *mer*-[Ru(bqp)(bqp-COOEt)]$(PF_6)_2$ (Ru-bqp-ester)

Purified *mer*-[Ru(bqp)(bqpCOOH)]$(PF_6)_2$ (100 mg) was suspended in 10 mL of ethanol and treated with three drops of concentrated sulfuric acid. The mixture was heated at reflux overnight, then cooled and poured onto 100 mL of an aqueous NH_4PF_6 solution to precipitate *mer*-[Ru(bqp)(bqpCOOEt)]$(PF_6)_2$ in quantitative yield. ^1H NMR (600 MHz, Acetone-d_6) δ (ppm): 8.46 (d, 2H), 8.43 (d, 2 H), 8.38 (s, 2 H), 8.35 (t, 1 H), 8.31 (d, 4 H), 8.15 (d, 2 H), 8.03 (d, 2 H), 7.98 (d, 2 H), 7.89 (dd, 4 H), 7.61 (m, 4 H), 7.27 (q, 4 H), 4.47 (m, 2 H), 1.39 (t, 3 H).

Synthesis of Ru-bqp Peptide Conjugates

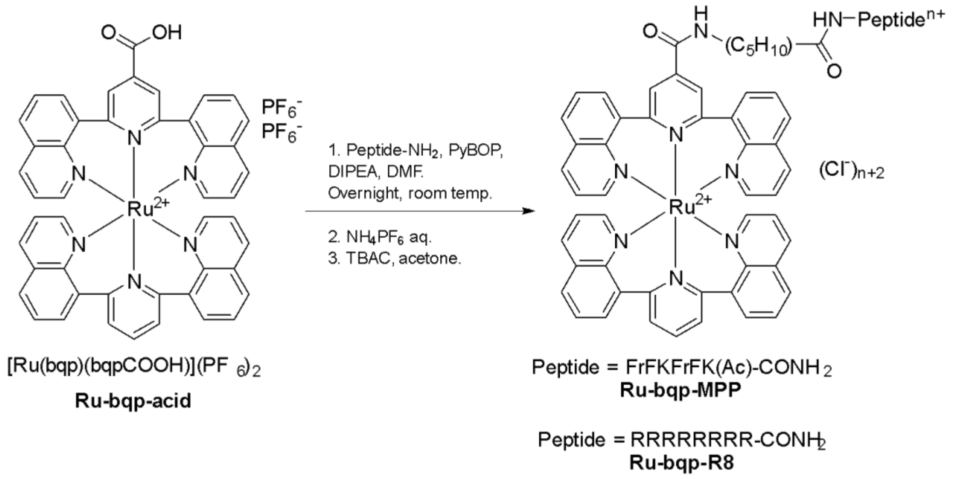

Scheme 1 – Peptide conjugation to Ru-bqp-acid to provide Ru-bqp-MPP and Ru-bqp-R8.Peptide conjugation was carried out using PyBOP coupling chemistry. Specifically, Ru-bqp-acid (16 mg, 1 eq.), peptide-NH_2 (40mg, about 2eq.), PyBOP (30 mg, 3 eq.) and DIPEA (20 uL), were stirred overnight in DMF (1 mL) in a glass vial. Next, the mixture was added dropwise to a stirring aqueous solution of NH_4PF_6 to precipitate the conjugate. The solid was filtered, washed well with water and allowed to dry. The crude material was dissolved in minimum acetone, filtered, and added dropwise to a stirring solution of tetrabutylammonium chloride in acetone. The precipitated chloride salt of the conjugate was collected by filtration and washed well with acetone.

[Ru-bqp-MPP], [Ru(bqp)(bqp-CONH-ahx-FrFKFrFK(Ac)-$CONH_2$]$^{5+}$.
^{1}H NMR (600 MHz, CD_3OD/D_2O) δ (ppm): 8.28 (m, 3H), 8.17 (m, 4H); 8.12 (m, 4H); 8.02 (m, 2H); 7.96 (m, 2H); 7.87 (d, 2H); 7.78 (m, 4H); 7.54 (m, 4H); 7.00 – 7.33 (m, 24H); 4.51 (m, 4H, peptide alpha-H); 3.80 – 4.30 (m, 4H, peptide alpha-H); 2.80 – 3.25 (m, 17H, peptide-H); 0.80 – 2.05 (m, 37H, peptide-H).
^{13}C NMR (CD_3OD/D_2O) δ (ppm): 122.69, 123.07, 127.52, 127.58, 127.65, 127.91, 128.61, 128.81, 131.06, 131.51, 132.77, 133.32, 133.82, 133.94, 138.37, 138.43, 138.96, 147.49, 147.57, 157.22, 157.81, 159.19, 159.62, 166.48.
HRMS (Q-Exactive, ESI(+)-qTOF, CH_3OH/TFA), *m/z* (Calculated, Assignment): 1175.9556 (1175.9559, [M - H^+ + 2TFA^-]$^{2+}$); 746.3077 (746.3087, [M - H^+ + TFA^-]$^{3+}$); 531.4844 (531.4852, [M – H^+]$^{4+}$); 425.3891 (425.3896, [M]$^{5+}$).

[Ru-bqp-R8], [Ru(bqp)(bqp-CONH-ahx-RRRRRRRR-$CONH_2$]$^{10+}$.
^{1}H NMR (600 MHz, CD_3OD/D_2O); very broad peaks, 8.17 – 8.42 (11 H), 8.03 - 8.14 (4 H), 7.90 - 7.97 (2 H), 7.80 – 7.89 (4 H), 7.58 – 7.67 (4 H), 7.18 – 7.28 (4 H), 4.41 (peptide alpha-H, 8 H), 3.53 (3 H), 0.95 – 2.50 (47 H, peptide-H).
HRMS (Q-Exactive, ESI(+)-qTOF, CH_3OH/TFA) *m/z (Calculated, Assignment)*: 714.7584 (714.7567, [M + 6TFA^-]$^{4+}$); 435.2167 (435.2154, [M – 5H^+]$^{5+}$); 362.8484 (363.8474, [M – 4H^+]$^{6+}$); 311.1567 (311.1559, [M – 3H^+]$^{7+}$).

Characterisation Data

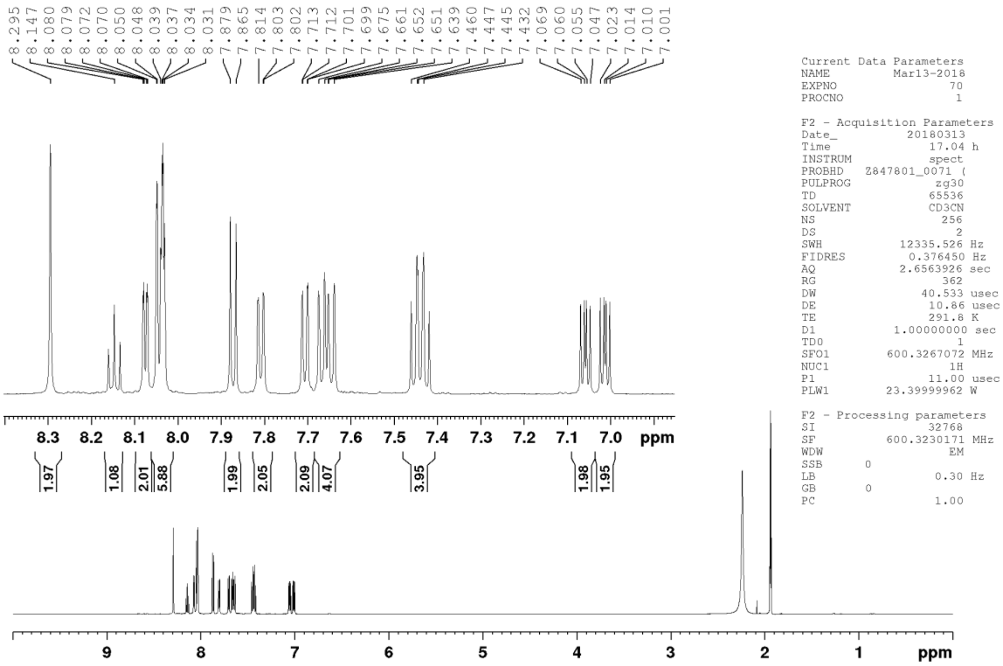

Figure S1. ^{1}H NMR Spectrum (600 MHz, CD_3CN) for *mer*-[Ru(bqp)(bqpCOOH)]$(PF_6)_2$ with insets to show regions of interest above the full spectrum.

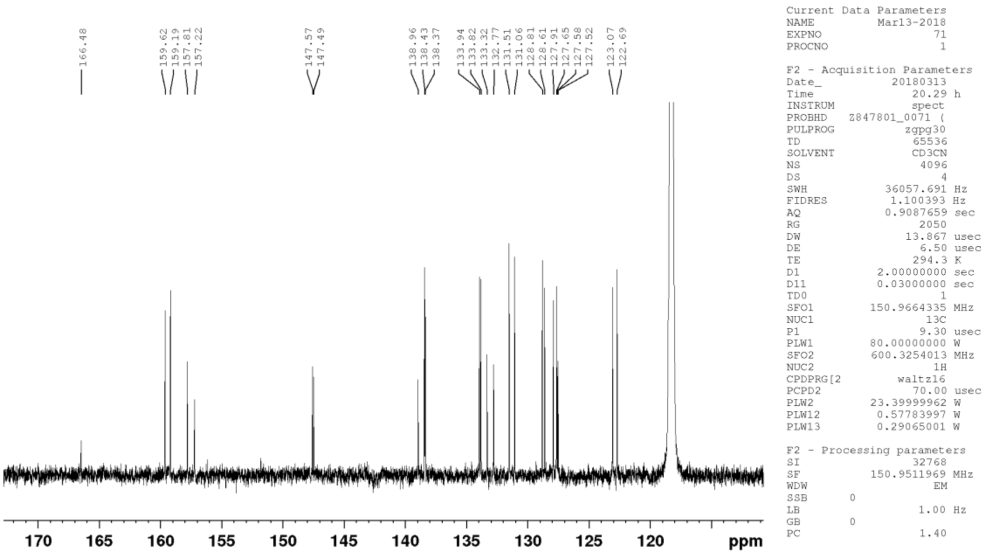

Figure S2. ^{13}C NMR Spectrum (150 MHz, CD_3CN) for *mer*-[Ru(bqp)(bqpCOOH)]$(PF_6)_2$ to show region of interest.

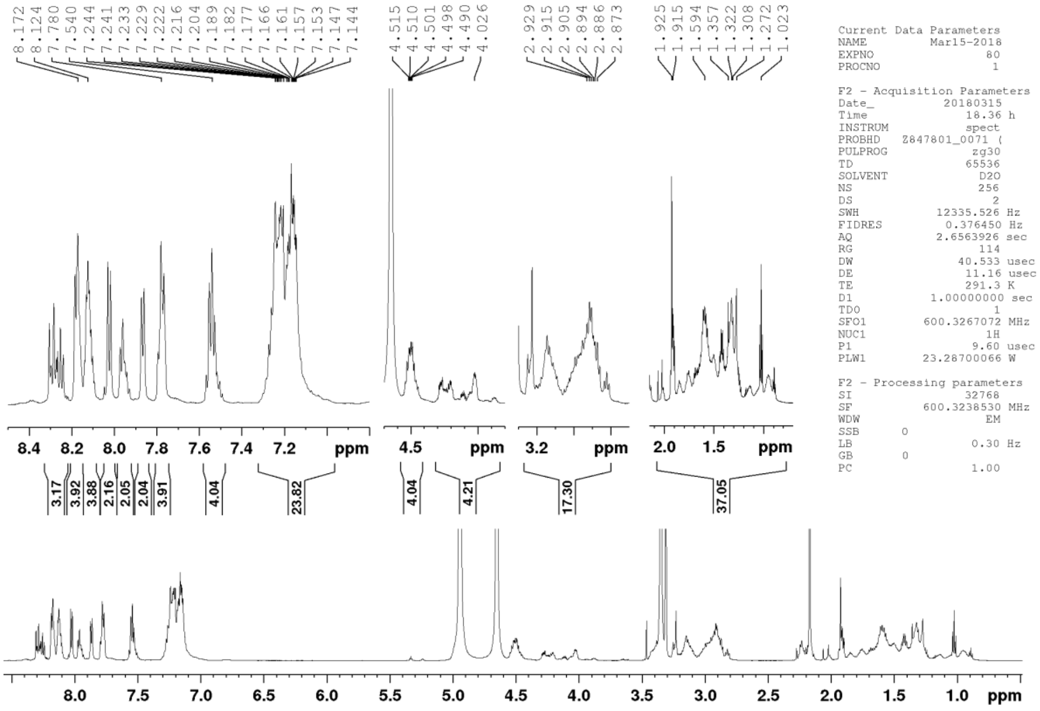

Figure S3. ^{1}H NMR Spectrum (600 MHz, CD_3OD/D_2O) for Ru-bqp-MPP with insets to show regions of interest above the full spectrum.

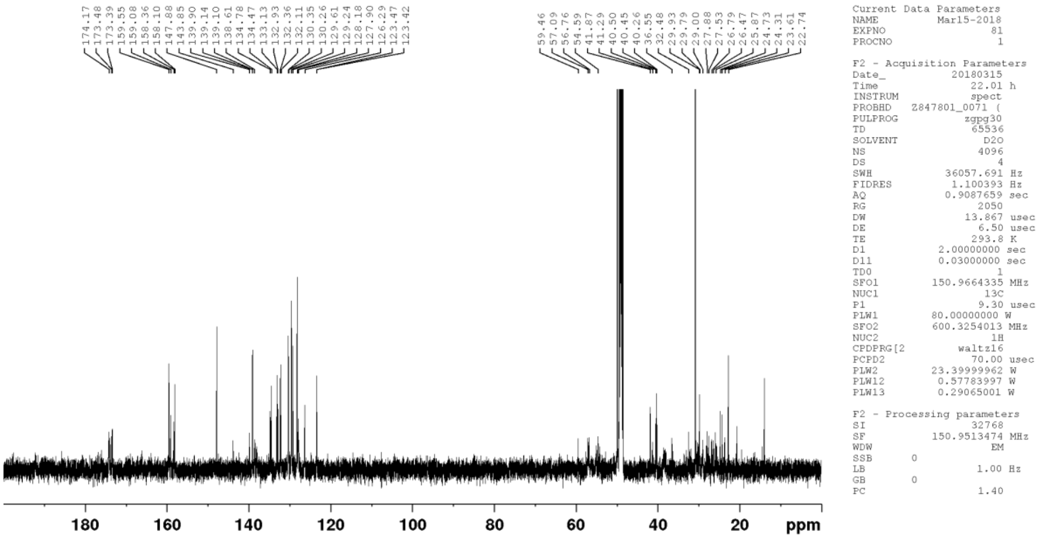

Figure S4. ^{13}C NMR Spectrum (150 MHz, CD_3OD/D_2O) for Ru-bqp-MPP.

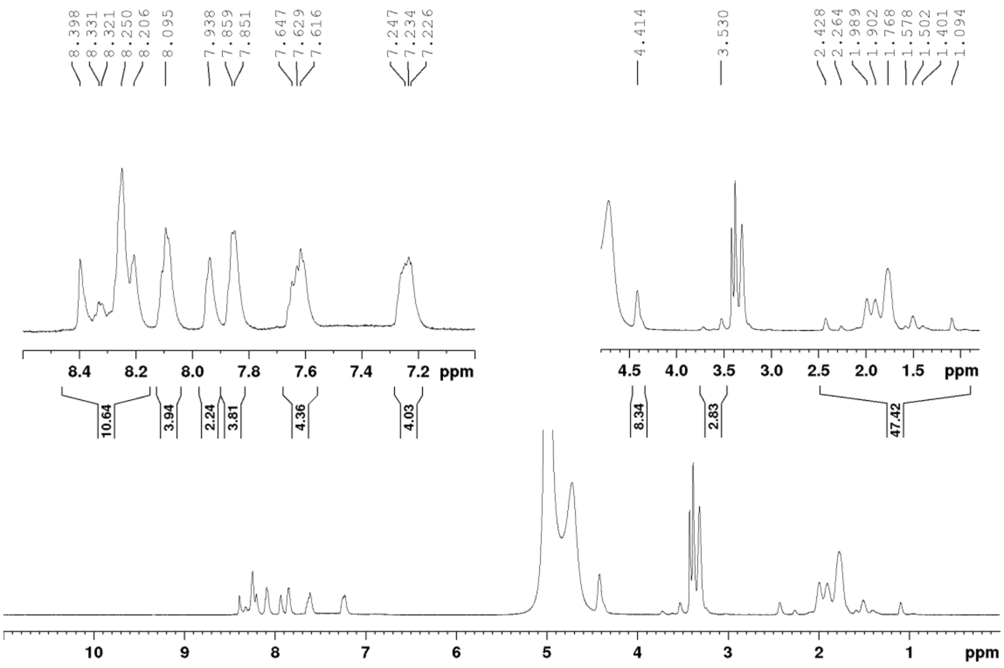

Figure S5. ^{1}H NMR Spectrum (600 MHz, CD_3OD/D_2O) for Ru-bqp-R8 with insets to show regions of interest above the full spectrum.

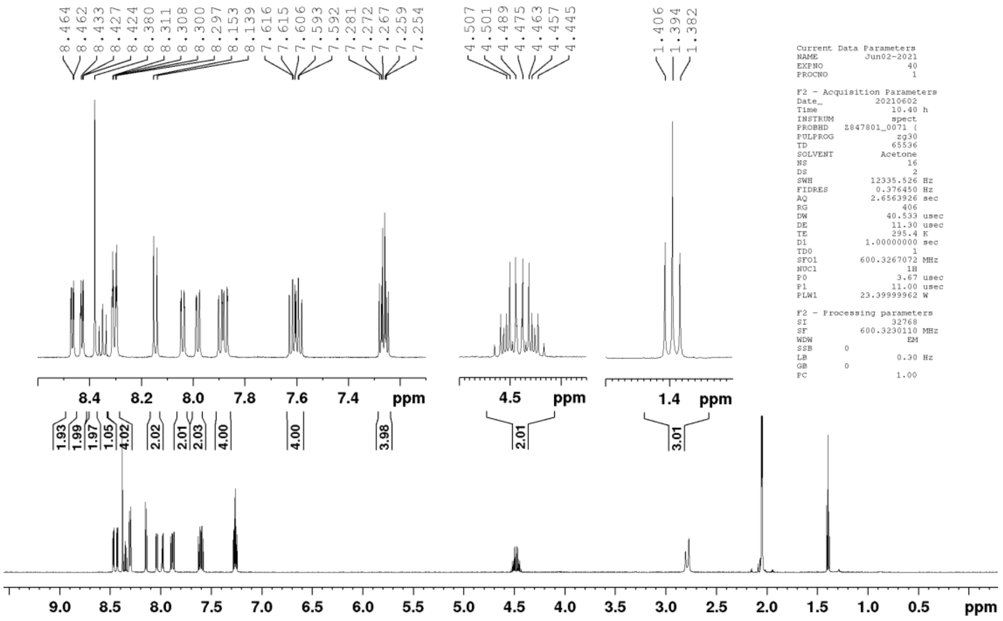

Figure S6. ^{1}H NMR Spectrum (600 MHz, Acetone-d_6) for *mer*-[Ru(bqp)(bqpCOOEt)]$(PF_6)_2$ with insets to show regions of interest above the full spectrum.

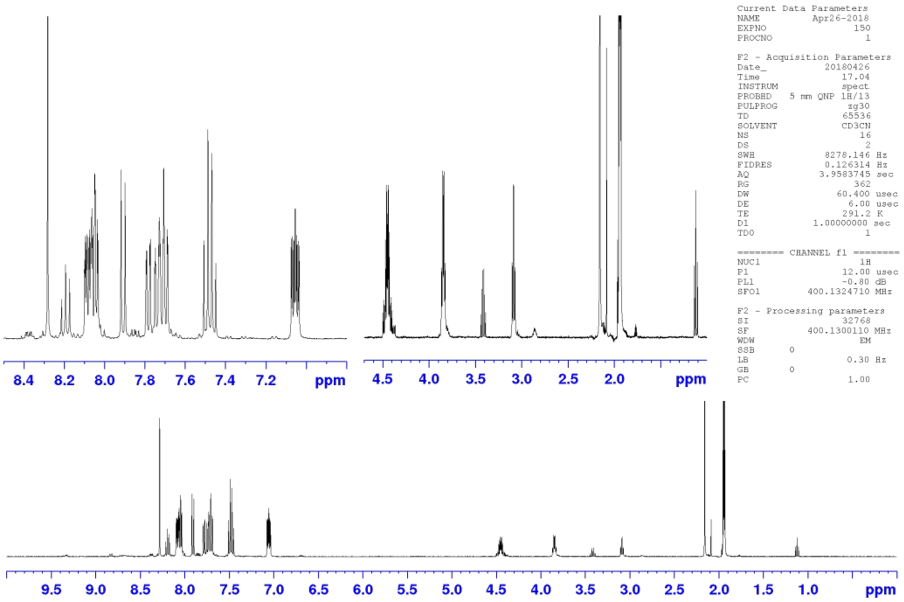

Figure S7. ^{1}H NMR Spectrum (400 MHz, CD_3CN) for the mixed ester *mer*-[Ru(bqp)(bqp-'ester')]$(PF_6)_2$ product obtained from long reflux in ethylene glycol with insets to show regions of interest above the full spectrum.

Single Mass Analysis
Tolerance = 50.0 PPM / DBE: min = -1.5, max = 200.0
Element prediction: Off
Number of isotope peaks used for i-FIT = 5

Monoisotopic Mass, Odd and Even Electron Ions
75 formula(e) evaluated with 1 results within limits (up to 10 best isotopic matches for each mass)
Elements Used:
C: 0-47 H: 0-30 N: 0-6 O: 0-2 Ru: 0-2

Christopher Burke (TK), CBTK0603
Q-TOF20180618MF020 47 (1.125) AM (Cen,6, 80.00, Ht,10000.0,1570.68,0.70); Sm (SG, 2x3.00); Sb (15,10.00); Cm (7:87-46:78) TOF MS LD+ 7.60e+002

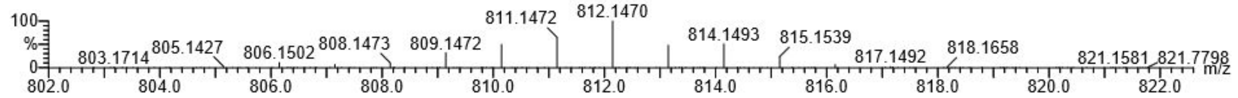

Minimum:				-1.5			
Maximum:		5.0	50.0	200.0			
Mass	Calc. Mass	mDa	PPM	DBE	i-FIT	i-FIT (Norm)	Formula
812.1470	812.1474	-0.4	-0.5	36.0	106.1	0.0	C47 H30 N6 O2 Ru

Figure S8. HR-MS (ESI-qTOF, MS+) spectrum for Ru-bqp-COOH to show region of interest and single mass analysis.

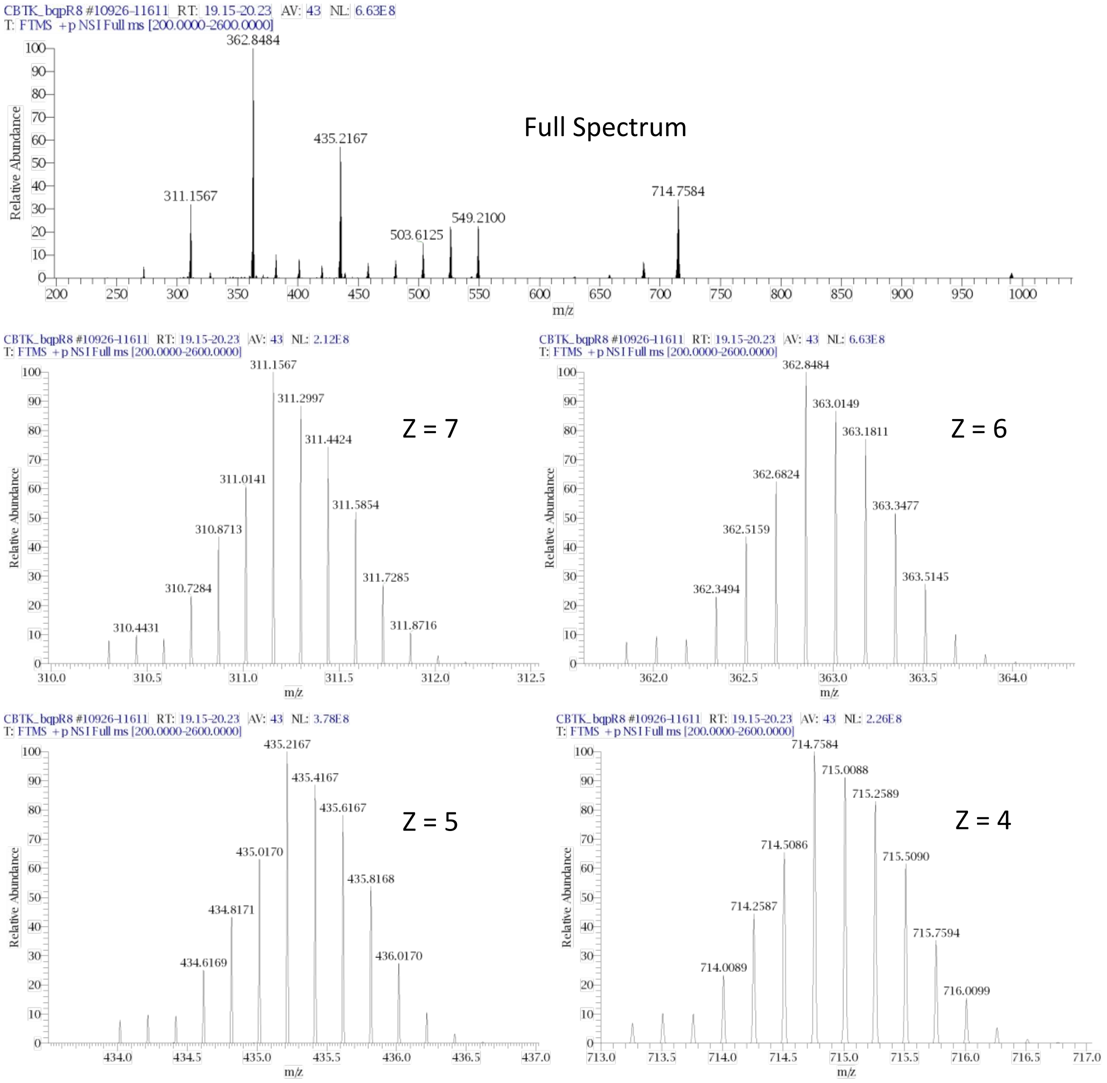

Figure S9. HR-MS analysis (Q-Exactive, Ion-Trap MS+, MeOH/TFA) for Ru-bqp-R8 to show the full spectrum at top and a close-up of individual major peaks with the associated ion calculated charge, Z.

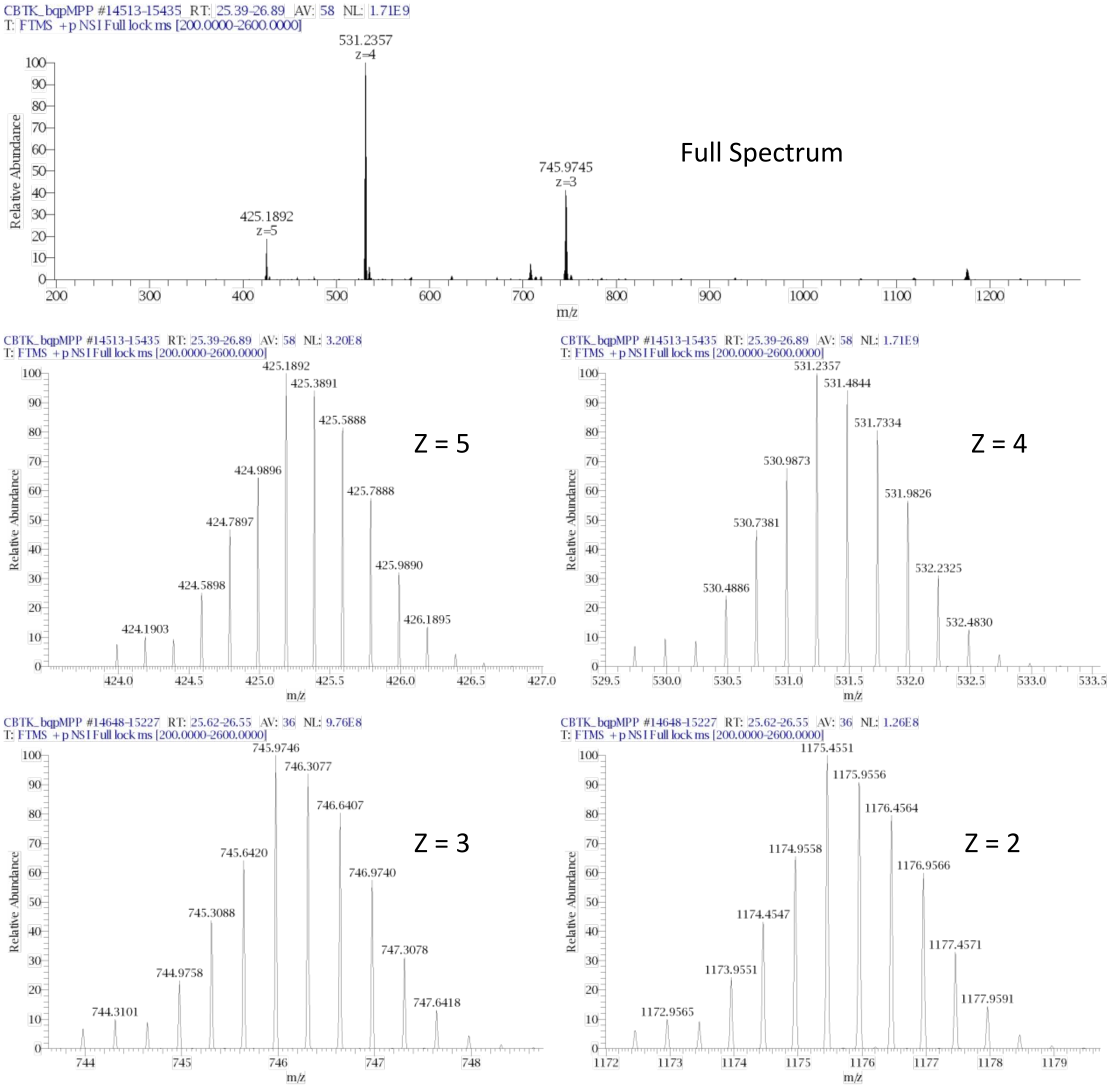

-

Figure S10. HR-MS analysis (Q-Exactive, Ion-Trap MS+, MeOH/TFA) for Ru-bqp-MPP to show the full spectrum at top and a close-up of individual major peaks with the associated ion calculated charge, Z.

Photophysical Studies

O_2 Sensitivity Studies

Oxygen sensitivity study results using Ru-bqp-MPP as an example:

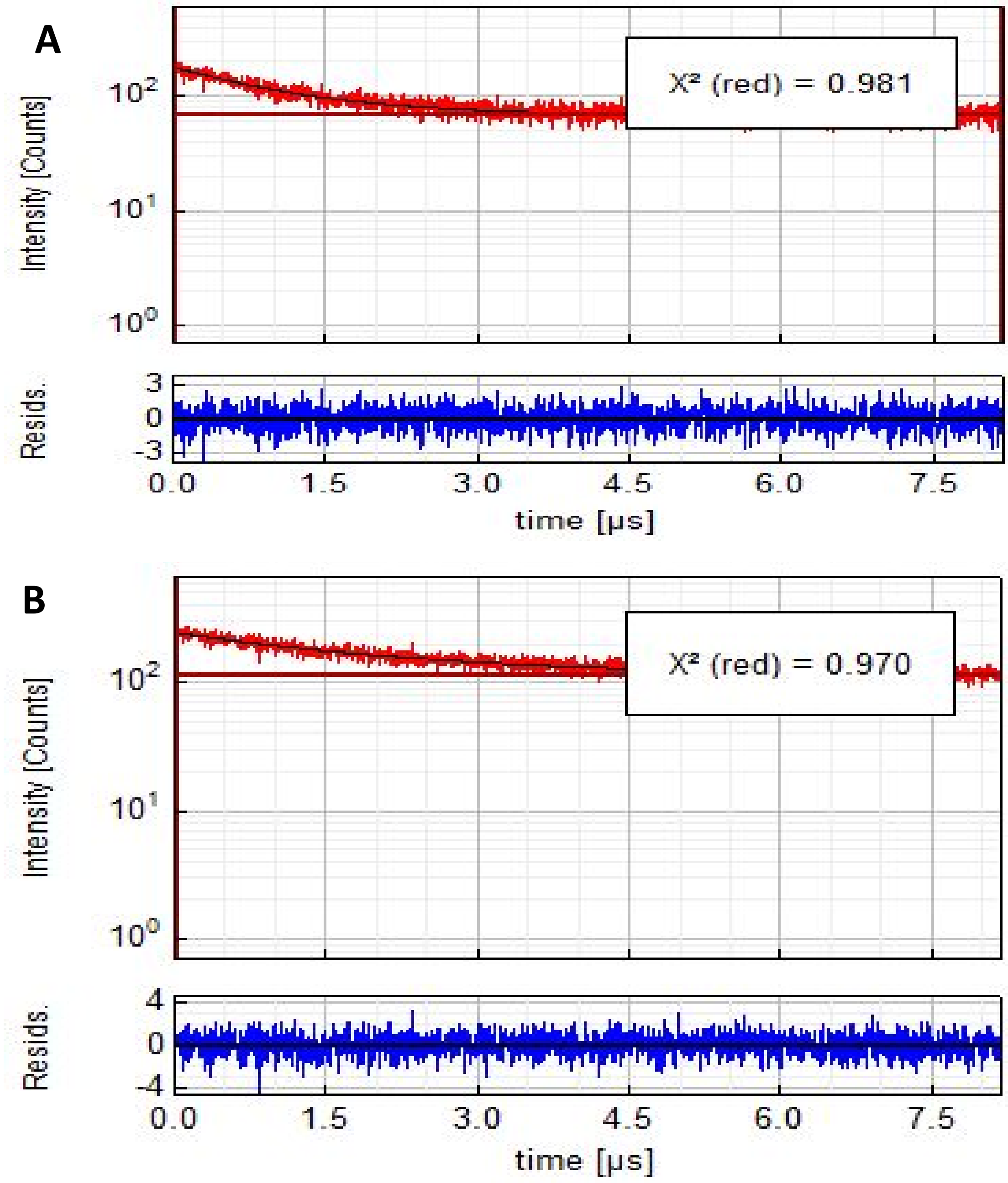

Figure S11. Emission decays of Ru-bqp-MPP (10 µM in PBS, pH 7.4) in A) aerated and B) de-aerated conditions. Aerated lifetime of 1.077 ± 0.033 µs and de-aerated lifetime 2.011 ± 0.021 µs exactly. Residual plots for the exponential fitting of the curves are shown below each plot.

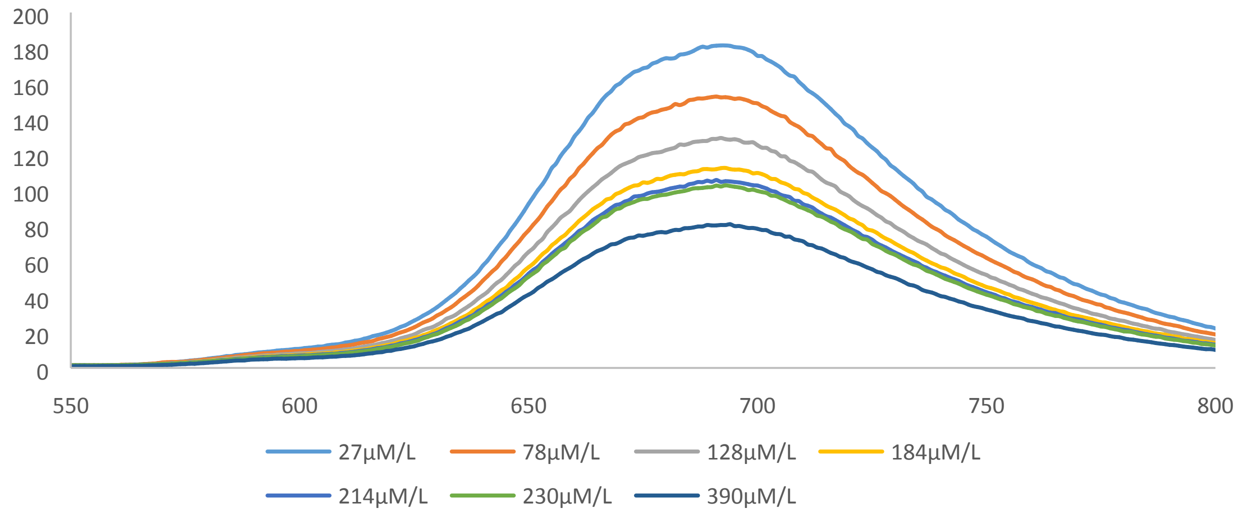

Figure S12. Ru-bqp-MPP emission intensity response to changing O_2 concentration (µM/L). Ru-bqp-MPP was excited at 494 nm with excitation and emission slit width of 10. O_2 concentration of 10 µM Ru-bqp-MPP in PBS (pH 7.4) was measured at room temperature.

Cell Studies

Confocal Laser Scanning Microscopy (CLSM)

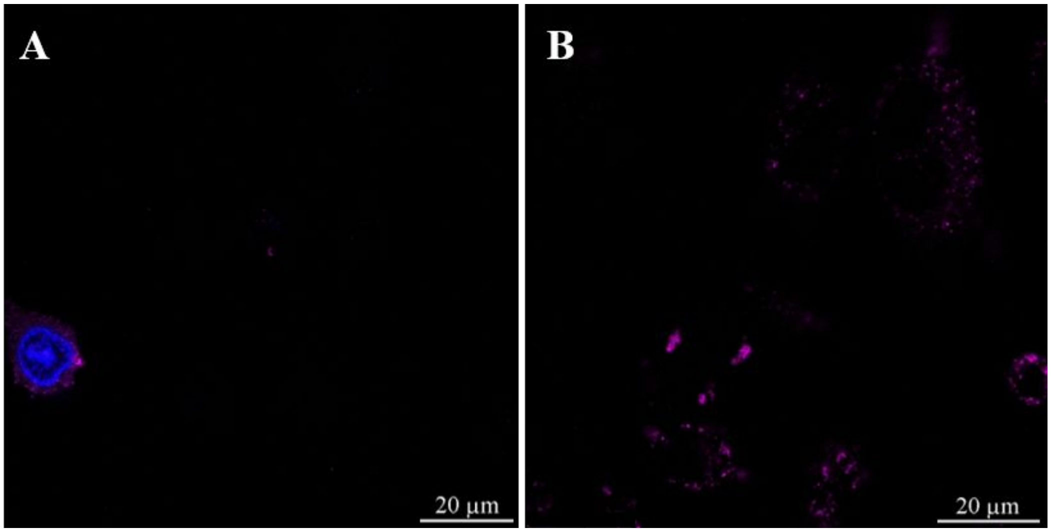

Figure S13. Ru-bqp-MPP (30 µM) at 3 h incubation in (A) A549 and (B) CHO cells. DRAQ7 (3 µM) was added to the cells before imaging to detect cell death. Ru-bqp-MPP is only seen in dead cells stained with DRAQ7 but remains in CHO under our regular imaging conditions including laser intensity.

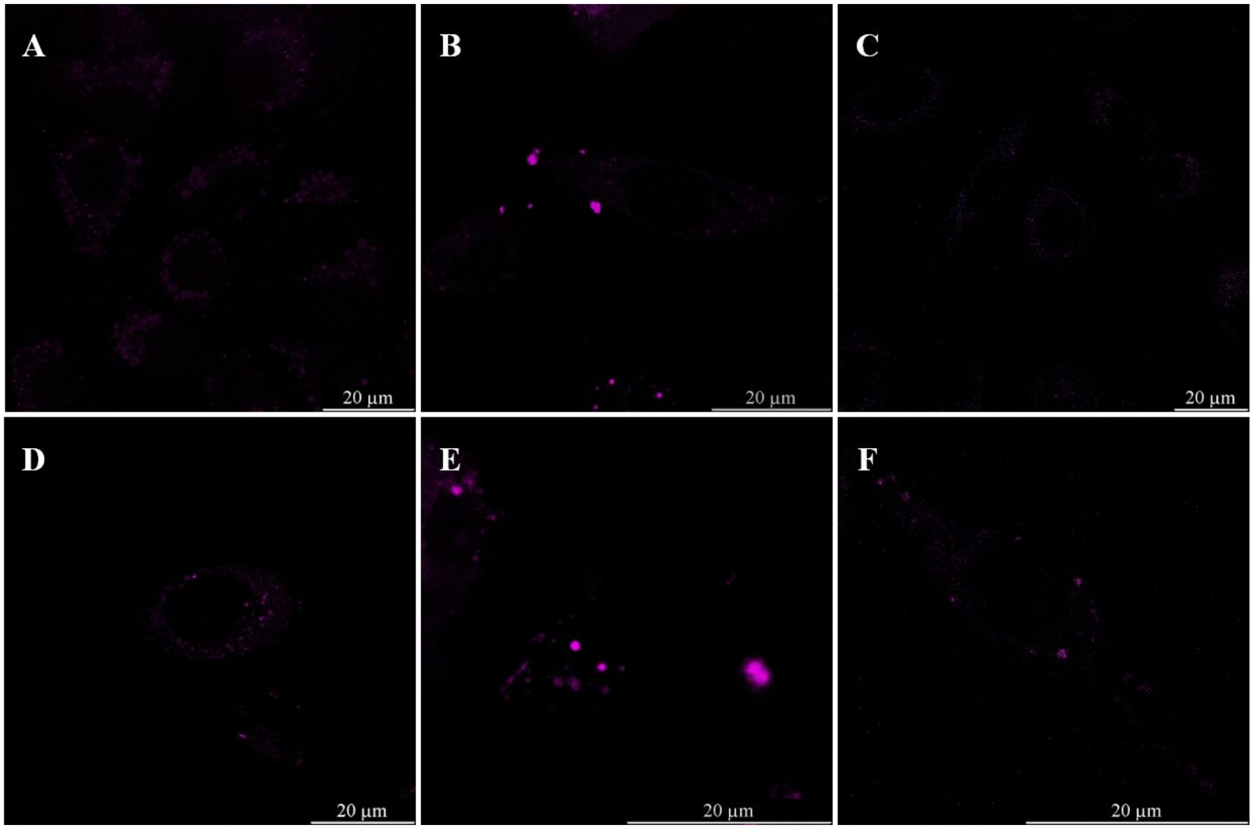

Figure S14. Cellular uptake of Ru-bqp-MPP in (A) A549 and (B) CHO cells, Ru-bqp-R8 in (B) A549 and (E) CHO cells and Ru-bqp-Ester in (C) A549 and (F) CHO cells. 30 μM and 1 h incubation with Ex 490 and Em 580-730 nm.

Localization Studies in 2D Cells

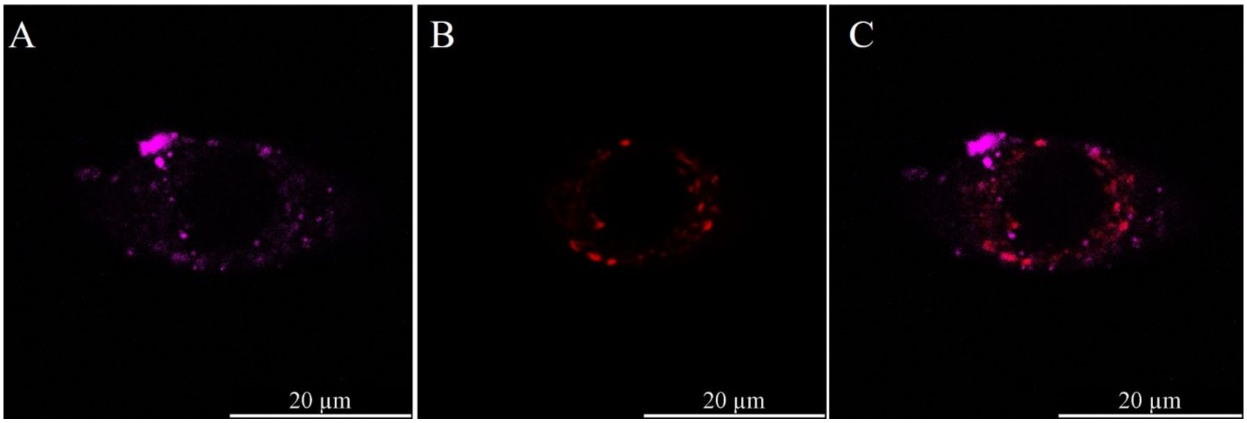

Figure S15. Cellular uptake and localization studies of Ru-bqp-R8 (30 μM) in live A549 cells. Cells were incubated for 1 h in the dark with (A) Ru-bqp-R8 and co-stained with (B) MitoTracker Deep Red (100 nM) for 30 minutes. Pearson's coefficient = 0.163 ± 0.02. A 470 nm white light laser was used to excite Ru-bqp-R8 and emission was collected between 580 and 700 nm. The MitoTracker Deep Red dye was excited at 644 nm and emission collected between 730-820 nm.

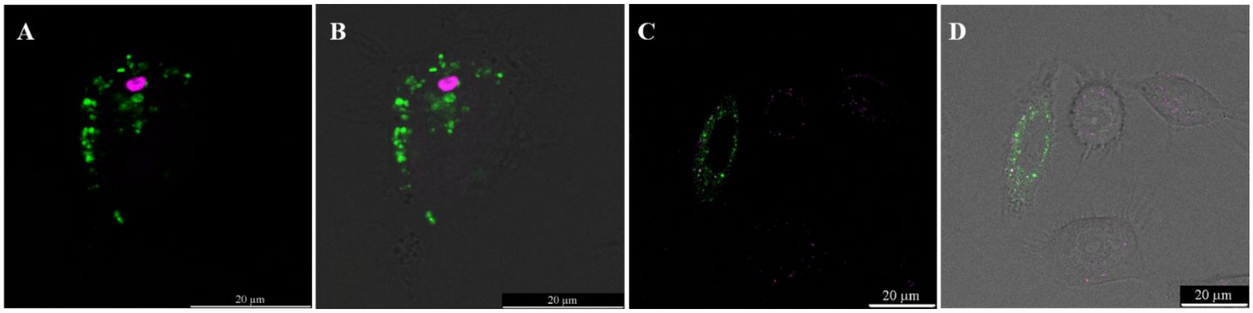

Figure S16. Co-localization studies of Ru-bqp-MPP (30 uM, 1 h) in live A549 (A-B) and CHO (C-D) cells. Co-localization with LysoTracker Green (Ex 504 nm Em 500-540 nm) is shown in (A) with brightfield overlay (B) and co-localization with the late endosomal dye Rab7a-GFP (C) with brightfield overlay (D). Co-localization values were obtained using specific regions of interest, as not all cells were stained with Rab7a-GFP.

Toxicity

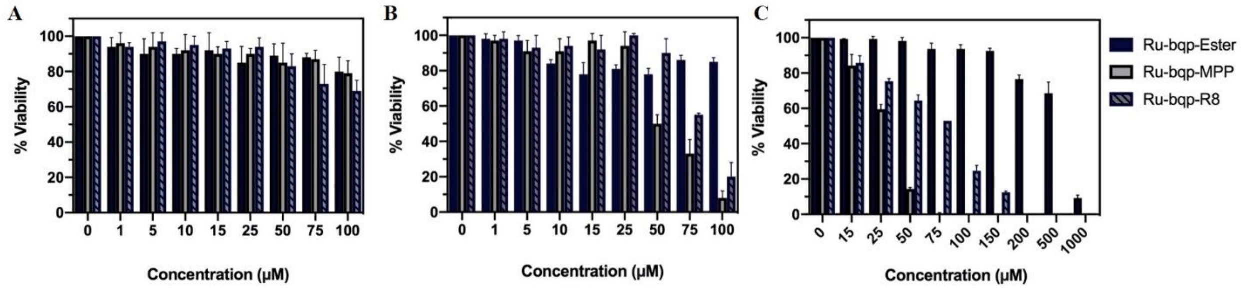

Figure S17. Cell viability of **(A)** A549, **(B)** CHO and **(C)** HPAC cells at 24 h exposure to varying concentrations of Ru-bqp-Ester, Ru-bqp-R8 and Ru-bqp-MPP in the absence of light. Cell viability was assessed using the Alamar Blue assay. Viability is determined as a percentage of untreated control cells incubated in media only. (N=3).

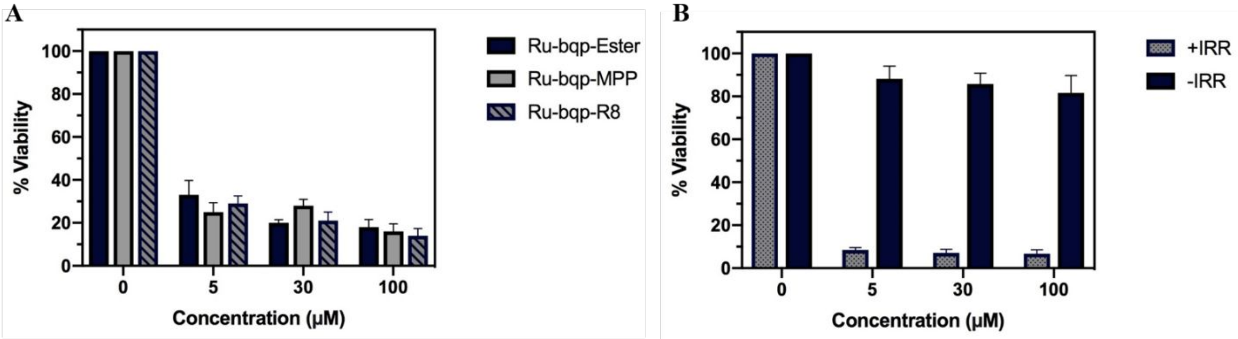

Figure S18. Cell viability of (A) A549 cells after a 30 minute incubation with Ru-bqp-Ester, Ru-bqp-MPP and Ru-bqp-R8 at 5, 30 and 100 µM followed by irradiation with a 470 nm blue light LED at 17 J/cm^2 (A549 dark control not graphed, cell viability > 80% up to 100 µM) and (B) CHO cells after 30 minute incubation with Ru-bqp-Ester at 5, 30 and 100 µM and irradiation (+IRR) at 17 J/cm^2 including CHO dark control (-IRR). Cell viability was assessed as per the cytotoxicity protocol described in experimental.

Table S1. Singlet oxygen ($^1O_2$) scavenger assay results. The decrease in absorbance at 410 nm is displayed as a percentage compared to the absorbance of the solution prior to irradiation (t=0) with a 470 nm LED.

Irradiation Time (minutes)	DPBF (10 mM)	DPBF (10 mM) with Ru-bqp-Ester (20 µM)	
	OD at 410 nm (DPBF only)	OD at 410 nm (Ru-DPBF)	% Decrease (compared to t=0)
**0**	3.118	3.047	0
**1**	2.995	2.916	4.30
**2**	2.916	2.572	15.59
**3**	2.867	0.965	68.33
**4**	3.035	0.947	68.92
**5**	2.847	0.613	79.88
**6**	2.194	0.630	79.32

3D Cell Studies

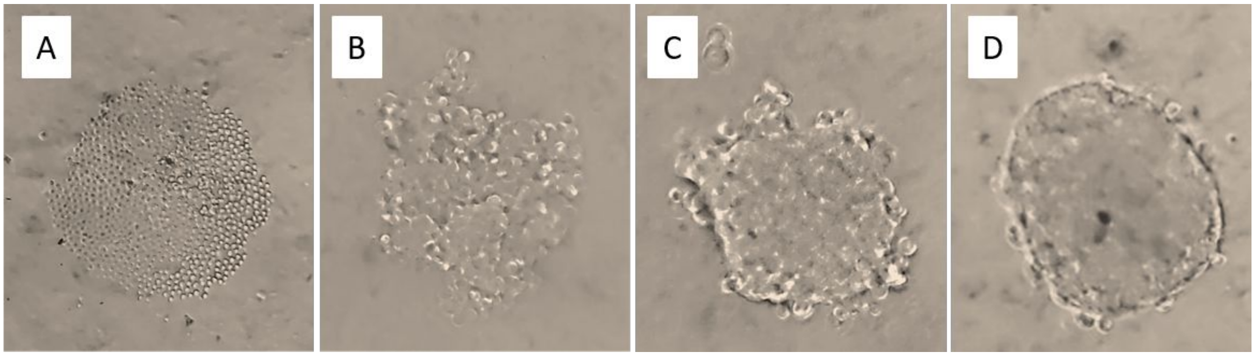

Figure S19. HPAC spheroid growth over 96 hours, imaged through an inverted Nikon (TMS) phase contrast light microscope focused using a 20x lens with 1x android camera magnification.

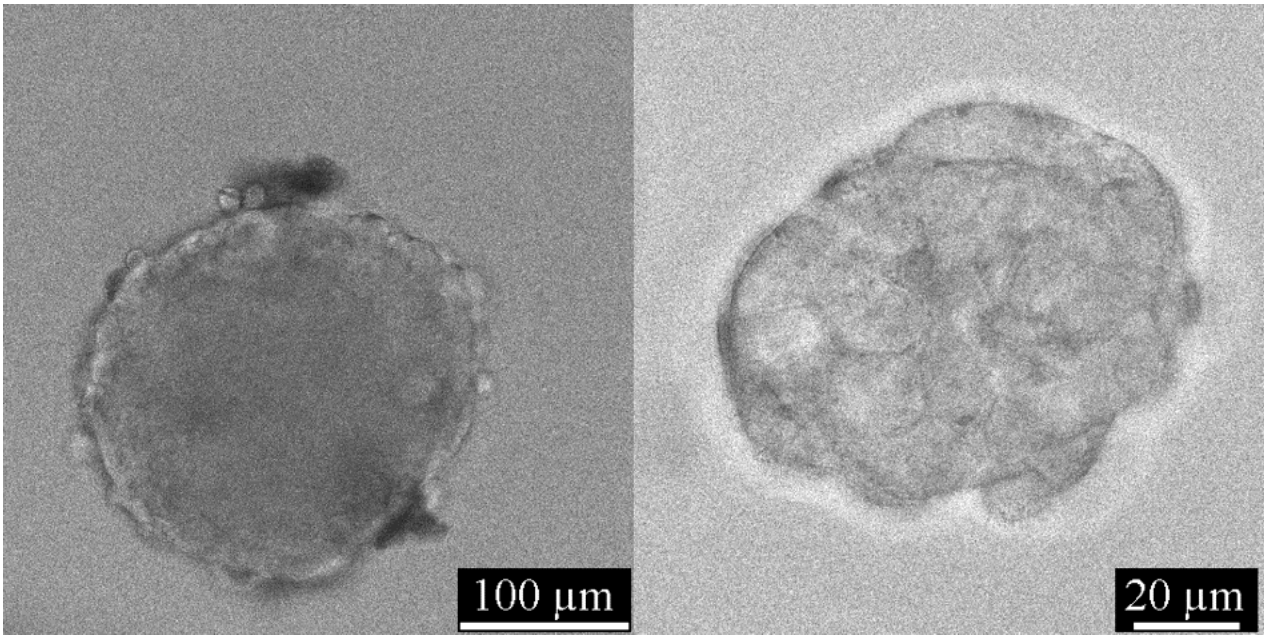

Figure S20. Live HPAC spheroids imaged using a confocal microscope (40x lens) after 96 hours growth.

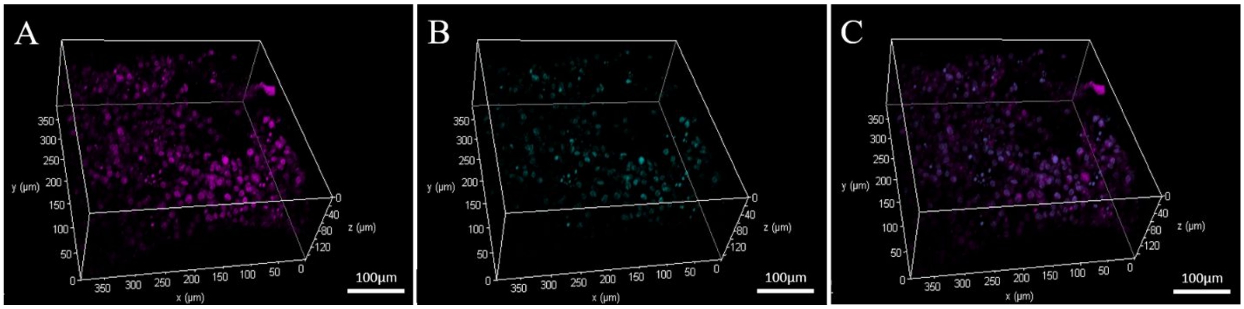

Figure S21. 3D reconstruction of a live HPAC spheroid with 100 µM Ru-bqp-MPP at 24 h and 1 µg/mL Hoechst 33342 nuclear stain added 45 minutes prior to imaging. Hoechst was excited using a 405 nm laser and emission collected between 425-475 nm, Ru-bqp-MPP was excited using a white light laser at 490 nm with emission 580-800 nm. Scale bar reads 100 µm.

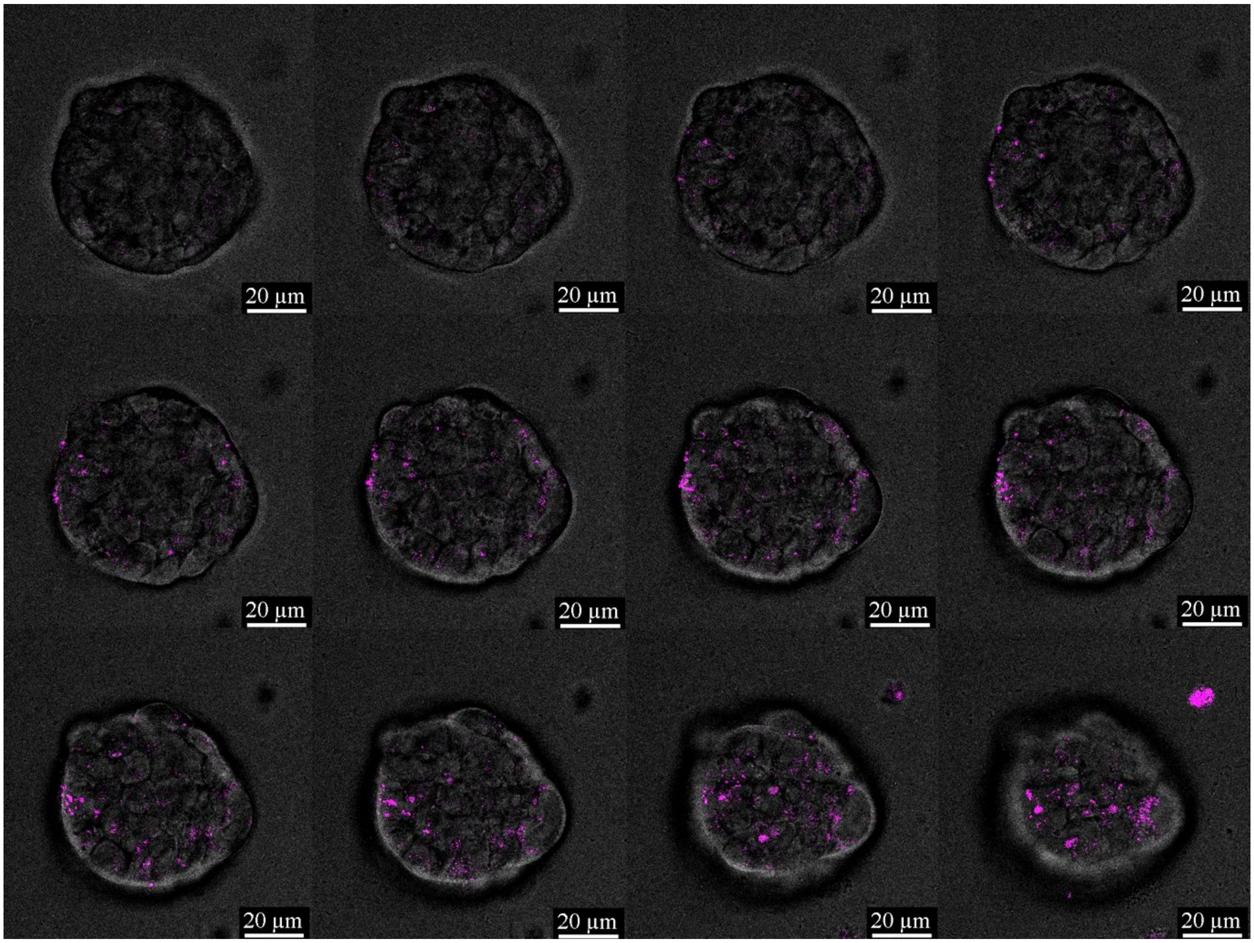

Figure S22. Z-stack images of Ru-bqp-MPP uptake in a HPAC spheroid (100 µM, 24 h) with brightfield contrast image overlay. Ex 490 nm & Em 580-800 nm (40x oil immersion lens).

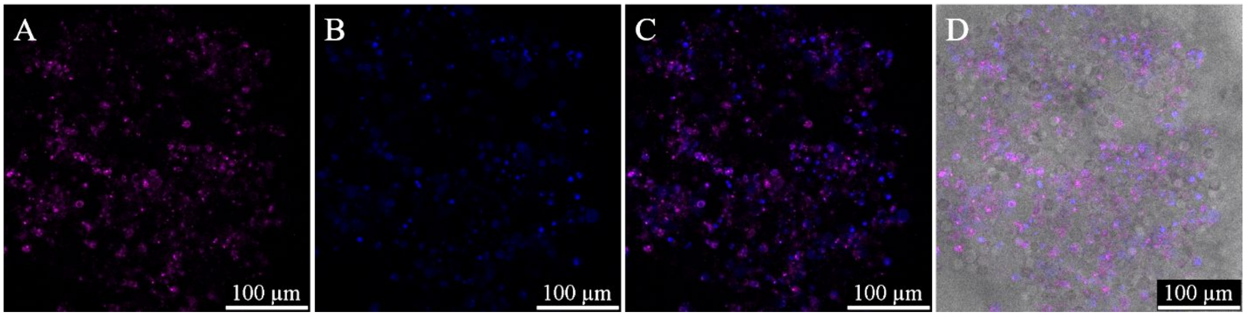

Figure S23. Individual z-stack image of the centre of a live HPAC spheroid with (A) 100 µM Ru-bqp-MPP, (B) 3 µM DRAQ7, (C) an overlay of B & C and (D) overlay with brightfield contrast image of the spheroid, imaged immediately after irradiation with a 470 nm LED (4.27 ± 0.41 J/cm^2). DRAQ7 uptake is linked to cellular death, but also cell damage. Spheroids were incubated overnight after irradiation to allow for cell recovery from radiation damage, control spheroids were shown to recover well.

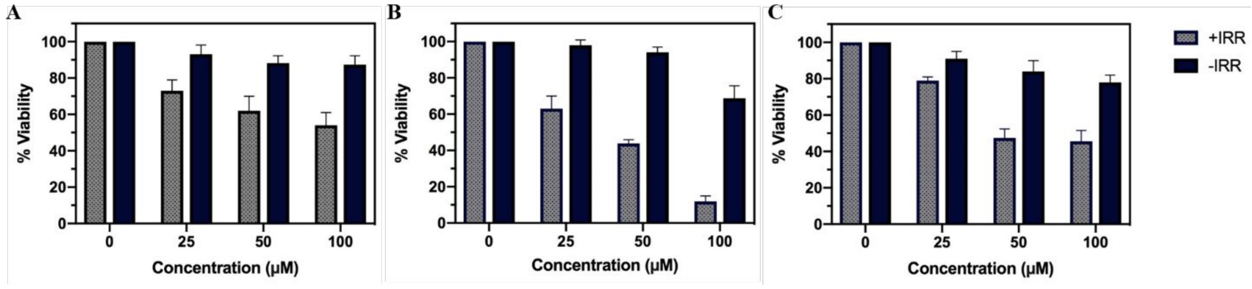

Figure S24. Dark and phototoxicity of our Ru(II) complexes in HPAC spheroids. The spheroids were incubated with **(A)** Ru-bqp-Ester, **(B)** Ru-bqp-MPP and **(C)** Ru-bqp-R8 at 25, 50 and 100 µM for 24 h and irradiated with a 470 nm blue light LED at 4.27 ± 0.41 J/cm^2. Dark control plates were performed alongside the phototoxicity assay and the dark and light IC_{50} values used to determine the PI values. Cell viability was assessed using the CellTiter-Glo 3D assay (N=3).
